# Composition and structure of Rosaceae leaf cuticles: insights into crystal formation and secondary alcohol biosynthesis

**DOI:** 10.1093/aob/mcaf308

**Published:** 2025-12-02

**Authors:** Christopher Cote, Reinhard Jetter, Abel Rosado

**Affiliations:** Department of Botany, University of British Columbia, Vancouver, BC V6T 1Z4, Canada; Department of Botany, University of British Columbia, Vancouver, BC V6T 1Z4, Canada; Department of Chemistry, University of British Columbia, Vancouver, BC V6T 1Z1, Canada; Department of Botany, University of British Columbia, Vancouver, BC V6T 1Z4, Canada

**Keywords:** cuticular wax, epicuticular wax crystals, nanotubes, gas chromatography, Rosaceae, SEM, secondary alcohols, 10-nonacosanol

## Abstract

**Background and Aims:**

The cuticle covers and protects aerial plant tissues from biotic and abiotic stressors, due in part to its unique composition of cuticular wax compounds and the presence of epicuticular wax crystals. The shape of these crystals is known to be dictated by specific compounds dominating the wax mixture. This study aimed to elucidate the chemical basis for such structures and understand the underlying wax biosynthetic mechanisms in two Rosaceae subfamilies: Amygdaloideae and Rosoideae.

**Methods:**

Gas chromatography coupled with mass spectrometry and flame ionization detection was used to characterize and quantify the leaf wax constituents of 12 species across two Rosaceae subfamilies. Scanning electron microscopy was used to investigate the leaf surface micromorphology.

**Key Results:**

Total wax amounts varied considerably within and between subfamilies, as did the presence of epicuticular wax crystals. Wax tubules were found exclusively in Amygdaloideae and composed primarily of 10-nonacosanol, whereas elongated, irregular platelets occurred only in Rosoideae and were composed of C_31_ and C_33_ aliphatics. Isomer analysis showed that secondary alcohols across subfamilies had a conserved 1:2 asymmetry, with hydroxyl groups on even- and odd-numbered carbons, demonstrating shared biochemistry. Amygdaloideae species primarily accumulated alkane pathway products, namely secondary alcohols. Rosoideae species accumulated more primary alcohol pathway products, and more alkanes than secondary alcohols.

**Conclusions:**

Although preferred biosynthetic mechanisms and wax structures are broadly grouped by subfamily, this organization breaks down at lower taxonomic ranks. In Rosaceae, cuticular biochemistry and structure may be quickly tuned within evolutionary time, reflecting yet unknown functional optimization of the cuticle.

## INTRODUCTION

The aerial organs of land plants are covered by a protective film called the cuticle, which evolved about 450 million years ago (Raven, 1977). Cutin and cuticular wax form this lipophilic layer, which functions as a permeability barrier, reducing non-stomatal water loss and protecting underlying tissues against environmental stress (Burghardt and Riederer, 2003). The specialized role of the cuticle stems from the unique chemistry and arrangement of its cuticular waxes (Bargel *et al.*, 2006; Jetter *et al.*, 2006), which vary widely across plant lineages.

Chemically, cuticular wax is a complex mixture dominated by very-long-chain (VLC) fatty acids (VLCFAs) and their derivatives, including aldehydes, alkanes, primary and secondary alcohols, ketones and esters, as well as secondary metabolites like pentacyclic triterpenoids (Jetter *et al.*, 2006; Samuels *et al.*, 2008). These compounds arise from the concerted activities of fatty acid synthase (FAS) and fatty acid elongase (FAE) complexes, which produce acyl-CoA precursors for all VLCFA-derived wax constituents, and differential elongation by the FAE gives rise to various chain-length homologues. All resulting VLC acyl-CoAs are then modified by two parallel alkane- and alcohol-forming pathways. In the alkane-forming pathway, VLCFAs are decarbonylated to alkanes, which may subsequently be oxidized to secondary alcohols, diols and ketones with functional groups on adjacent even- and odd-numbered carbons, as demonstrated in *Arabidopsis thaliana* (Greer *et al.*, 2007; Wen and Jetter, 2009). Alternatively, secondary alcohols may be synthesized via condensation of functionalized acyl-CoAs, leading to hydroxyl groups on even-numbered carbons only (Busta and Jetter, 2018). In the alcohol-forming pathway, VLCFAs are reduced to primary alcohols, which may condense with long-chain fatty acyls, giving rise to alkyl esters (Samuels *et al.*, 2008). Although the core biosynthetic machinery is conserved across land plants, the relative abundance, chain-length distributions and compound class profiles of wax mixtures are strongly shaped by developmental and environmental factors (Jetter and Schäffer, 2001; Shepherd and Wynne Griffiths, 2006).

From a structural standpoint, cuticular wax consists of an intracuticular fraction, embedded within the cutin polymer matrix, and an epicuticular fraction, which lies on the exterior surface of cutin (Buschhaus and Jetter, 2011). Waxes in the latter fraction may self-assemble at the cuticle surface into crystalline projections known as epicuticular wax crystals (EWCs). EWCs govern surface properties such as hydrophobicity, self-cleaning and UV protection (Jeffree *et al.*, 1975; Mulroy, 1979; Neinhuis and Barthlott, 1997; Koch *et al.*, 2009), and variations in their composition give rise to various morphologies (Jeffree *et al.*, 1976). Thus, EWC shapes range from well-defined structures such as tubules and platelets to less-ordered deposits like wax crusts and smooth layers (Barthlott *et al.*, 1998). Among the various EWC morphologies, tubules are one of the best understood chemically. These elongated, hollow projections are strongly associated with high concentrations of either β-diketones or 10-nonacosanol, a C_29_ secondary alcohol bearing a hydroxyl group at the C-10 position (Barthlott *et al.*, 1998). Co-crystallization of 10-nonacosanol with C_29_ diols, with one hydroxyl group located at C-10 and another positioned on various other carbons along the chain, is proposed to stabilize tubule growth (Jetter and Riederer, 1995). Compositional analyses coupled with microscopy support this mechanism, establishing a clear chemistry–structure relationship for these EWCs. However, a significant knowledge gap remains regarding the chemical determinants of other prevalent EWC morphologies, such as triangular rodlets. This gap stems primarily from the historical separation of morphological and compositional analyses, where scanning electron microscopy (SEM) studies often document crystal structures without accompanying chemical characterization, and vice versa.

The Rosaceae family offers a suitable comparative framework for elucidating the relationship between wax composition and crystal morphology, as it comprises two well-defined subfamilies, Amygdaloideae and Rosoideae, which diverged ∼100 million years ago (Xiang *et al.*, 2017). Moreover, these subfamilies display distinct EWC morphologies (Neinhuis and Barthlott, 1997; Tomaszewski and Zieliński, 2014), where tubules are observed exclusively in Amygdaloideae, triangular rodlets predominantly in Rosoideae, and smooth layers or wax crusts in both subfamilies (Neinhuis and Barthlott, 1997; Tomaszewski and Zieliński, 2014). This evolutionary split sets an ideal stage for examining how different wax compositions give rise to subfamily-specific EWC forms.

Chemical studies within Rosaceae have focused primarily on commercially important Amygdaloideae species, such as *Prunus laurocerasus* (Jetter *et al.*, 2000; Jetter and Schäffer, 2001; Diarte *et al.*, 2021), *Prunus avium* (Peschel *et al.*, 2007; Hunsche and Noga, 2011), and *Malus domestica* (Huang, 2017; Chai *et al.*, 2020). Although 10-nonacosanol has been identified in the wax mixtures of various Amygdaloideae species, including *Amelanchier alnifolia* (Knowles *et al.*, 1996), *Malus domestica* (Huang, 2017) and *Prunus domestica* (Ismail *et al.*, 1976), its specific role in tubule formation remains unconfirmed within this family. In contrast, members of the Rosoideae subfamily remain less studied, though wax profiles have been characterized in *Rosa* (Buschhaus *et al.*, 2007; Cheng *et al.*, 2019) and, to a limited extent, in *Fragari*a (Baker and Hunt, 1979; Jiang *et al.*, 2024) species. Thus, despite their consistent occurrence across multiple genera within Rosoideae, no compositional basis has been proposed for triangular rodlets. This imbalanced research focus has left critical gaps in our understanding of wax diversity across this family.

The present study explores the chemical and structural diversity of the cuticular waxes among diverse Rosaceae genera that are native or naturalized to coastal British Columbia, Canada. Focusing on species from a single geographic region across Amygdaloideae and Rosoideae subfamilies minimizes environmental impacts on wax accumulation while maximizing phylogenetic diversity. We aim to establish the chemical determinants of the observed subfamily-specific EWC morphologies through integrated compositional and micromorphological analyses, and to advance our understanding of wax biosynthesis in rosaceous plants.

## MATERIALS AND METHODS

### Plant material

Fully expanded leaves were sampled in September 2023 from plants growing at the University of British Columbia (UBC) Botanical Garden. Five replicates per species were prepared and stored for up to 1 month at 4 °C in polystyrene or polyethylene containers before wax extraction (Table 1). Extracted leaf surface area was determined using ImageJ™ with calibrated photographs of fresh material (Supplementary Data Table S1).

**Table 1.** List of species included in the investigation, including subfamily and tribal classification in Rosaceae (Xiang *et al.*, 201*7*), leaf types, number of leaves or leaflets per replicate, and number of individuals sampled.

Subfamily	Tribe	Species	Simple (S) or compound (C) leaf	No. of leaves/leaflets per replicate	No. of individuals sampled
Amygdaloideae	Amygdaleae	*Prunus emarginata*	S	1	5^a^
	Exochordeae	*Oemleria cerasiformis*	S	1	4
	Maleae	*Amelanchier alnifolia*	S	1–3	4
		*Malus fusca*	S	1–3	5
	Spiraeae	*Aruncus dioicus*	C	1	5
		*Spiraea lucida*	S	1–3	5^a^
Rosoideae	Agrimonieae	*Sanguisorba canadensis*	C	1	3
	Potentilleae	*Fragaria chiloensis*	C	3	5^a^
		*Potentilla indica*	C	3	5^a^
	Roseae	*Rosa nutkana*	C	3	2
	Rubeae	*Rubus armeniacus*	C	2	5
		*Rubus spectabilis*	C	2	5

^a^Individuals that were part of a clonal patch.

### Wax extraction

Leaf samples were placed in Erlenmeyer flasks and spiked with a known amount of tetracosane internal standard before being extracted three times with CHCl_3_ (99 %, 1 % ethanol stabilizer, Sigma–Aldrich, St Louis, MO, USA) for 30 s. Extracts were combined in 20-mL glass scintillation vials and concentrated under N_2_ flow (>99.998, Linde) at 70 °C and quantitatively transferred to 2-mL GC vials and further concentrated. Wax extracts were derivatized with 25 µL each of bis-*N*,*N*-(trimethylsilyl)trifluoroacetamide (BSTFA) (GC grade, Sigma–Aldrich) and pyridine (>99.8 %, anhydrous, Sigma–Aldrich) at 70 °C for 30 min, concentrated, and reconstituted in ∼1.5 mL of CHCl_3_.

### Wax analysis

Wax composition was determined using previously established methods (Zhang *et al.*, 2024), with qualitative analysis conducted using gas chromatography–mass spectrometry (GC–MS). One microlitre of the sample was injected using a cool-on-column injector (54 °C) and separated on a 6890 GC (Agilent, Santa Clara, CA, USA) equipped with an HP-1 capillary column (30 m length × 320 µm inner diameter × 0.1 µm film thickness) using He carrier gas (>99.995 %, Linde) flowing at 1.4 mL min^−1^. The oven was programmed to hold at 50 °C for 2 min, increase to 200 °C at 40 °C min^−1^, hold for 2 min, and increase to 320 °C at 3 °C min^−1^, where it was held for 30 min. Separated compounds were subsequently identified by comparison of spectra obtained with a 5793N Mass Selective Detector (Agilent, Electron impact 70 eV) with spectra of authentic standards. The relative amounts of secondary alcohol homologue isomers for *Amelanchier alnifolia* and *Fragaria chiloensis* were quantified using the average abundance of each isomer’s two characteristic α-fragments generated by GC–MS. Quantitative analysis of wax samples was conducted using an 8860 GC (Agilent) with the same GC parameters as above but using H_2_ as the carrier gas (>99.995 %, Linde) flowing at 2.0 mL min^−1^. Analytes were detected using a flame ionization detector (FID) (Agilent). Each compound’s wax load (µg cm^−2^) was determined by comparing its GC peak area against the internal standard peak area, divided by leaf surface area.

### Scanning electron microscopy

Abaxial and adaxial leaf samples were excised from mature leaves and mounted on stubs with adhesive (Zhang *et al.*, 2024). Samples were dried over desiccant at room temperature for 2 weeks and subsequently sputter-coated with ∼3 nm of gold/palladium using an Electron Microscope ACE600 (Leica, Wetzlar, Germany). Samples were imaged with aZeiss Crossbeam 350 ultrahigh-resolution scanning electron microscope (Zeiss, Oberkochen, Germany) at a 3 kV accelerating voltage.

### Phylogenetic analysis

Partial coding sequences of ribulose-1,5-bisphosphate carboxylase/oxygenase large subunit (*rbcL*) genes of the investigated species were obtained from the NCBI nucleotide database and aligned with the Clustal Omega multiple sequence alignment (MSA) tool (Madeira *et al.*, 2024) (Supplementary Data Table S2). The tree file generated from the MSA was converted to a cladogram in R using the ape (Paradis and Schliep, 2019) and ggtree packages (Yu, 2020).

### Statistical analysis

Analysis of variance (ANOVA) and Tukey’s honest significant difference (HSD) test were conducted in R to compare the means of quantitative wax traits between species. Principal component analysis (PCA) was used to analyse associations between species and normalized cuticular wax traits (e.g. relative compound class wax load) in R using the ggplot2 (Wickham, 2016) and ggfortify (Tang *et al.*, 2016) packages. Relative individual wax compound data were centred log-ratio (CLR) transformed and used to carry out an agglomerative hierarchical clustering analysis (HCA) of Rosaceae species in R.

## RESULTS

The current study aimed to advance our understanding of cuticular wax crystal formation and biosynthesis through comparative chemical and micromorphological analyses of Rosaceae leaf surfaces. Six species each from the Amygdaloideae and Rosoideae subfamilies were analysed by (1) quantifying total wax by GC–FID and observing the cuticle surface using SEM, (2) further quantifying specific wax compounds using GC, and (3) characterizing the isomer profiles of secondary alcohols within the mixtures via GC coupled with MS.

### Total wax amounts and epicuticular wax morphology

**Fig. 1.**
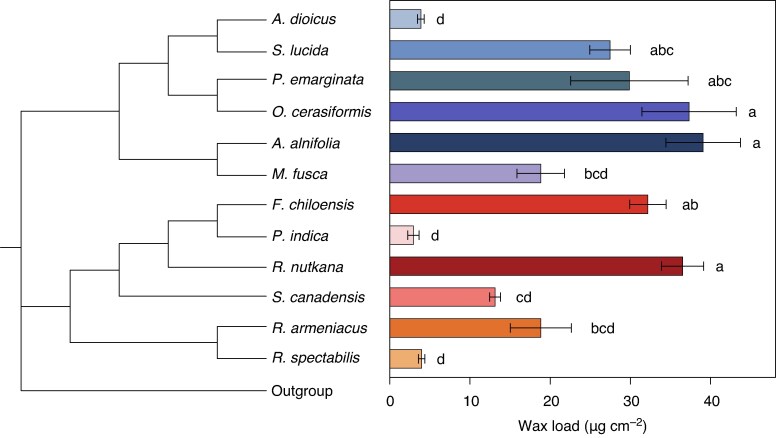
Total leaf wax load of 12 Rosaceae species. Coverage is expressed as micrograms of wax per square centimetre of leaf surface. Bars represent the mean of biological replicates (*n* = 5), and error bars represent the standard error. Different letters represent significantly different wax loads between species (*P* < 0.05, ANOVA). Species: *Aruncus dioicus*, *Spiraea lucida*, *Prunus emarginata*, *Oemleria cerasiformis*, *Amelanchier alnifolia*, *Malus fusca*, *Fragaria chiloensis*, *Potentilla indica*, *Rosa nutkana*, *Sanguisorba canadensis*, *Rubus armeniacus* and *Rubus spectabilis*.

First, we used GC–FID to quantify the total leaf cuticular wax loads. These amounts ranged between 4 and 40 µg cm^−2^ across Amygdaloideae species, and between 3 and 37 µg cm^−2^ across Rosoideae species (Fig. 1). Relatively large differences in total wax coverage were observed between pairs of phylogenetically related species in each subfamily (e.g. *Aruncus dioicus* vs *Spiraea lucida*, *Fragaria chiloensis* vs *Potentilla indica*).

**Fig. 2.**
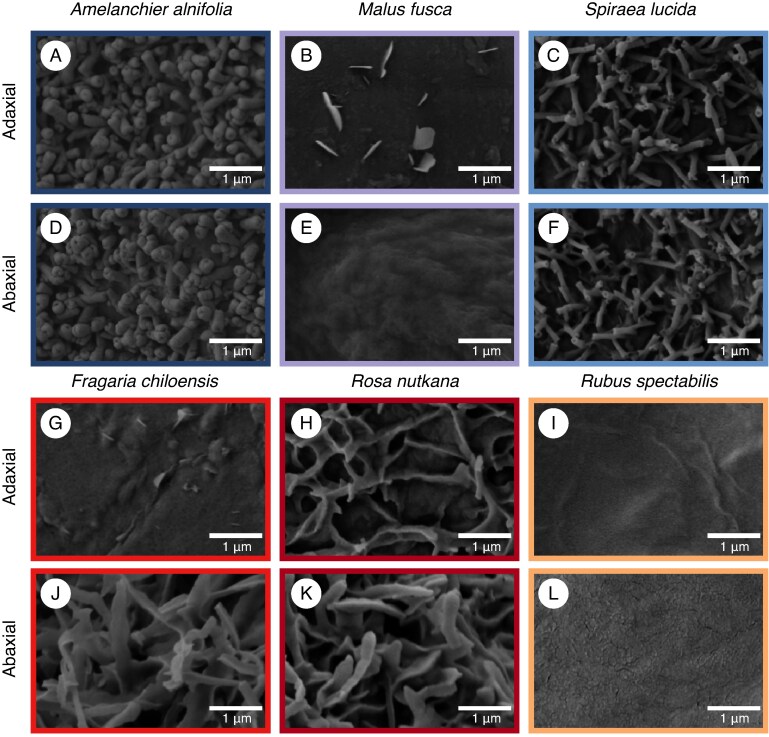
Scanning electron micrographs of six Rosaceae species’ leaf surfaces at ×10 000 magnification. Adaxial leaf surfaces of Amygdaloideae (A–C) and Rosoideae (G–I) species. Abaxial leaf surfaces of Amygdaloideae (D–F) and Rosoideae species (J–L).

Next, we analysed the morphology of epicuticular wax structures on abaxial and adaxial leaf surfaces of Rosaceae species using SEM. Here we present three representative species per subfamily in detail (Fig. 2), and the remaining six species, which show similar wax morphologies, in the supplementary information (Supplementary Data Fig. S1). The abaxial side of *Malus fusca* (Fig. 2B) and the adaxial side of *Rubus spectabilis* (Fig. 2I) leaves were covered with smooth epicuticular wax films, lacking surface texture. Similar patterns were observed on both sides of *Aruncus dioicus* (Supplementary Data Fig. S1A, D) and *Rubus armeniacus* leaves (Supplementary Data Fig. S1H, K), and the abaxial sides of *Prunus emarginata* (Supplementary Data Fig. S1F) and *Potentilla indica* (Supplementary Data Fig. S1J). The waxes on the abaxial side of *Rubus spectabilis* (Fig. 2L) leaves, as well as the adaxial sides of *Prunus emarginata* (Supplementary Data Fig. S1C) and *Potentilla indica* leaves (Supplementary Data Fig. S1G), appeared as crusts with irregular surface topography. Tubules with distinct pores were observed on both surfaces of *Amelanchier alnifolia*, *Spiraea lucida* and *Oemleria cerasiformis* leaves (Fig. 2A, D, C, F; Supplementary Data Fig. S1B, E). These tubules were ∼1 µm long in all three species, and their diameter ranged from ∼0.1 µm in *Spiraea lucida* to 0.25 µm in *Amelanchier alnifolia* and *Oemleria cerasiformis*. The tubules were distributed across the leaf surfaces, such that most of the outermost epicuticular film beneath them was obscured. *Malus fusca* leaves had upright wax platelets ranging from 0.25 to 0.75 µm in width on the adaxial surface (Fig. 2B). In contrast, elongated platelets with jagged margins were found on the leaves of *Rosa nutkana*, *Fragaria chiloensis* and *Sanguisorba canadensis* (Fig. 2H, J, K; Supplementary Data Fig. S1I, L), with underlying epicuticular film visible between them only on the adaxial surface of *Rosa nutkana* and on both sides of *Sanguisorba canadensis* leaves.

### Relative load of compound classes, chain lengths and individual wax compounds

**Fig. 3.**
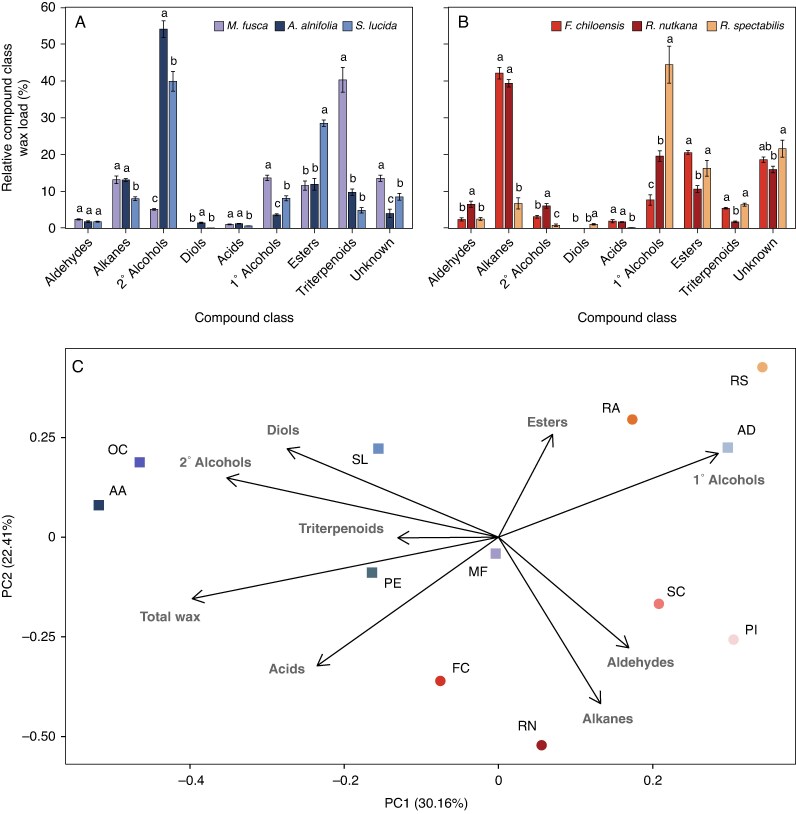
Compound class distribution in the leaf cuticular waxes of Rosaceae species. Relative amounts of compound classes in Amygdaloideae (A) and Rosoideae (B) species. Bars represent the mean of biological replicates (*n* = 5) and error bars represent standard error. Different letters indicate significant differences in compound class percentages among species (*P* < 0.05, ANOVA). (C) PCA based on relative loads of wax compound classes. Amygdaloideae species are represented by squares and abbreviated as follows: AA (*Amelanchier alnifolia*), AD (*Aruncus dioicus*), MF (*Malus fusca*), OC (*Oemleria cerasiformis*), PE (*Prunus emarginata*), SL (*Spiraea lucida*). Rosoideae species are represented by circles and abbreviated as follows: FC (*Fragaria chiloensis*), PI (*Potentilla indica*), RA (*Rubus armeniacus*), RN (*Rosa nutkana*), RS (*Rubus spectabilis*), SC (*Sanguisorba canadensis*).

To identify and quantify the chemical components of the Rosaceae leaf cuticular wax mixtures, we used GC–MS and GC–FID, respectively. Among the Amygdaloideae, secondary alcohols were the most abundant compound class in waxes of *Amelanchier alnifolia* (53 %) and *Spiraea lucida* (39 %). In contrast, *Malus fusca* leaf wax was predominantly composed of triterpenoids (38 %) (Fig. 3A; Supplementary Data Table S3). Aldehydes, alkanes, fatty acids and primary alcohols were present at ∼2–13 % across these three species. Alkyl esters represented 29 % of the total leaf wax in *Spiraea lucida,* and 12 % in *Amelanchier alnifolia* and *Malus fusca*. Diols constituted 1 and 0.1 % of the *Amelanchier alnifolia* and *Spiraea lucida* leaf waxes, respectively, but were not detected in *Malus fusca* wax. We found similar results for three additional Amygdaloideae species (Supplementary Data Fig. S2A).

Among the Rosoideae species, alkanes were present in high amounts in *Fragaria chiloensis* (39 %) and *Rosa nutkana* (37 %) leaf waxes but not in *Rubus spectabilis* (7 %) (Fig. 3B; Supplementary Data Table S3). Conversely, primary alcohols were the predominant compound class in *Rubus spectabilis* and present in lower concentrations in *Fragaria chiloensis* (8 %) and *Rosa nutkana* (20 %) leaf waxes. In all three species, aldehydes, secondary alcohols, fatty acids, alkyl esters and triterpenoids constituted between 1 and 19 % of the leaf wax mixtures. Only trace amounts of alkanediols were found in the respective Rosoideae wax mixtures. We found comparable compositions for three additional Rosoideae species (Supplementary Data Table S3; Supplementary Data Fig. S2B).

PCA was used to compare wax profiles and identify compound classes that capture the main variation in our data. The values for the mean relative amounts of the eight identified compound classes, along with total wax loads for each species, were input as variables. The first and second principal components, PC1 and PC2, explained 30.16 and 22.41 % of the variation, respectively (Fig. 3C). The amounts of total wax, triterpenoids, secondary alcohols, diols, primary alcohols and, to a lesser extent, acids comprised PC1, while the quantities of esters, alkanes and aldehydes comprised PC2. Given their shorter loading vectors, triterpenoids and esters minimally contributed to the variation encompassed by their respective PCs. Among the Amygdaloideae, *Amelanchier alnifolia*, *Spiraea lucida* and *Oemleria cerasiformis* were most closely associated with secondary alcohols and diols, *Prunus emarginata* with triterpenoids, and *Aruncus dioicus* with primary alcohols. In contrast, *Malus fusca* was not associated with a particular compound class. Among the Rosoideae, *Rubus spectabilis* and *Rubus armeniacus* were associated with esters and primary alcohols, *Sanguisorba canadensis* and *Potentilla indica* with aldehydes, and *Fragaria chiloensis* and *Rosa nutkana* with alkanes.

Additional GC–FID analysis of the wax mixtures revealed the chain length distributions across all the wax compound classes in the surveyed species. The various compound classes displayed similar patterns of odd and even carbon numbers between Amygdaloideae and Rosoideae wax mixtures. Only even-numbered aldehyde, acid and alkyl ester homologues were detected in all species, along with primary alcohols comprising mainly even-numbered chain lengths and traces of odd-numbered homologues (Supplementary Data Figs S3–S6). Odd-numbered homologues dominated alkanes and secondary alcohols, though even-numbered homologues were also detected in a few species. C_29_ was the only alkanediol chain length detected across all Rosaceae, with the isomer nonacosane-5,10-diol being the most prevalent (Supplementary Data Tables S4 and S5; Supplementary Data Fig. S7).

Within the Amygdaloideae, C_29_ secondary alcohol was the most abundant compound in *Amelanchier alnifolia* (49 %) and *Spiraea lucida* (35 %) leaf wax mixtures, alongside notable amounts of C_27_–C_31_ secondary alcohols (Supplementary Data Table S4). Additional prominent compounds included C_30_ aldehyde, C_29_ and C_31_ alkanes, and C_28_ acid. The dominant primary alcohol and ester homologue differed among Amygdaloideae species. Ursolic acid was the most abundant compound among the Amygdaloideae leaf wax triterpenoids, and comprised 30 % of the *Malus fusca* wax mixture, making it the most abundant single compound. Within the Rosoideae, C_31_ and C_33_ alkanes were the most abundant compounds in *Fragaria chiloensis* (15 and 21 %, respectively) and *Rosa nutkana* (15 and 16 %) leaf wax mixtures. In comparison, C_26_ alcohol was the most abundant compound in *Rubus spectabilis* (20 %) wax (Supplementary Data Table S5), and the most abundant primary alcohol homologue in *Fragaria chiloensis* and *Rosa nutkana* leaf waxes. Additionally, C_28_ was the predominant aldehyde homologue across the Rosoideae. The dominant acid and ester homologues differed among species. Uvaol was the most abundant triterpenoid in *Fragaria chiloensis* and *Rosa nutkana* leaf waxes, while β-amyrin was the most abundant triterpenoid in *Rubus spectabilis*.

**Fig. 4.**
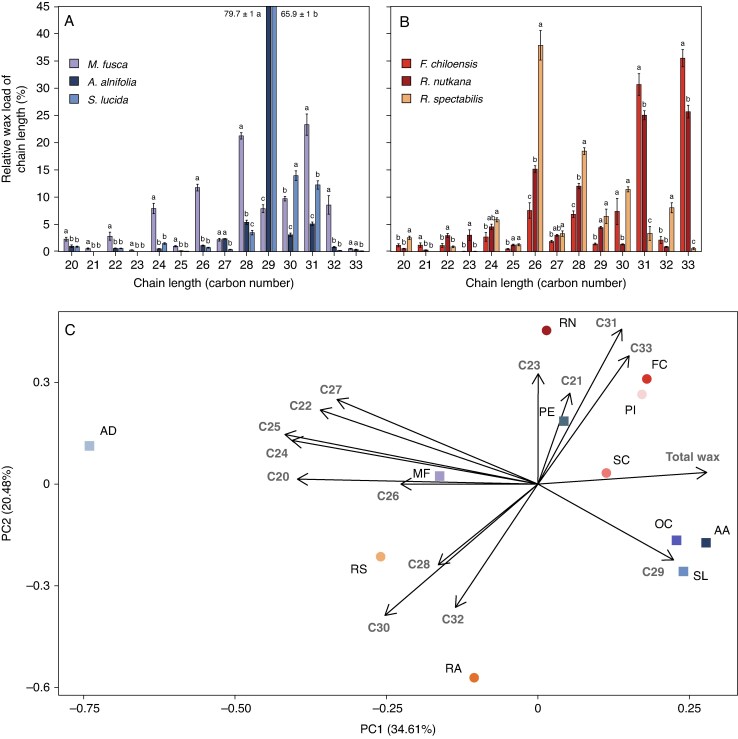
Overall chain length distribution of cuticular wax compounds of Rosaceae species. Relative chain length amounts summed across aldehydes, alkanes, secondary alcohols, diols, fatty acids and primary alcohols in the leaf wax mixtures of Amygdaloideae (A) and Rosoideae (B) species. Bars represent the mean of biological replicates (*n* = 5), and error bars represent the standard error. Different letters indicate significant differences in relative chain length wax loads among species (*P* < 0.05, ANOVA). (C) PCA based on relative loads of chain lengths. Amygdaloideae species are represented by squares and abbreviated as follows: AA (*Amelanchier alnifolia*), AD (*Aruncus dioicus*), MF (*Malus fusca*), OC (*Oemleria cerasiformis*), PE (*Prunus emarginata*), SL (*Spiraea lucida*). Rosoideae species are represented by circles and abbreviated as follows: FC (*Fragaria chiloensis*), PI (*Potentilla indica*), RA (*Rubus armeniacus*), RN (*Rosa nutkana*), RS (*Rubus spectabilis*), SC (*Sanguisorba canadensis*).

Finally, the overall chain length profile across all compound classes in the leaf wax from each species was analysed. Among the Amygdaloideae, *Amelanchier alnifolia* and *Spiraea lucida* had narrow chain length profiles centred at C_29_ that were otherwise not biased towards even or odd carbon numbers (Fig. 4A). In contrast, *Malus fusca* had a broad bimodal chain length distribution peaking at C_28_ and C_31_, with even chain lengths predominating up to C_28_ and similar amounts of even- and odd-numbered homologues around C_31_. In the Rosoideae, *Fragaria chiloensis* and *Rosa nutkana* had mainly even-numbered chain lengths up to C_30_, together with predominant C_31_ and C_33_ compounds (Fig. 4B). In contrast, *Rubus spectabilis* displayed a broader chain length profile centred on C_26_ and was overall skewed towards even chain lengths. Similar results for six additional Rosaceae species are reported (Supplementary Data Fig. S8).

PCA was used to identify further aliphatic compound chain lengths that contribute most strongly to the variation in the data, using the total wax amounts and mean relative chain length loads from C_20_ to C_33_ for each species as inputs (Fig. 4C). PC1 and PC2 explained 34.61 and 20.48 % of the dataset variation, respectively. Total wax, C_20_, C_22_, C_24_, C_25_, C_26_, C_27_ and C_29_ amounts strongly loaded on PC1, while the remaining chain lengths loaded on PC2. C_21_, C_23_, C_26_ and C_28_ contributed relatively little to the variation explained by their respective PCs. Among the Amygdaloideae, *Amelanchier alnifolia*, *Spiraea lucida* and *Oemleria cerasiformis* were most closely associated with C_29_, *Prunus emarginata* with C_31_, *Malus fusca* with C_26_, and *Aruncus dioicus* with several of the PC1 chain lengths. Among the Rosoideae, *Fragaria chiloensis* and *Potentilla indica* were closely associated with C_33_, *Rosa nutkana* with C_31_, *Rubus spectabilis* with C_28_, *Rubus armeniacus* with C_32_ and *Sanguisorba canadensis* with total leaf wax amount.

### *Amelanchier alnifolia* and *Fragaria chiloensis* secondary alcohol isomer distributions

**Fig. 5.**
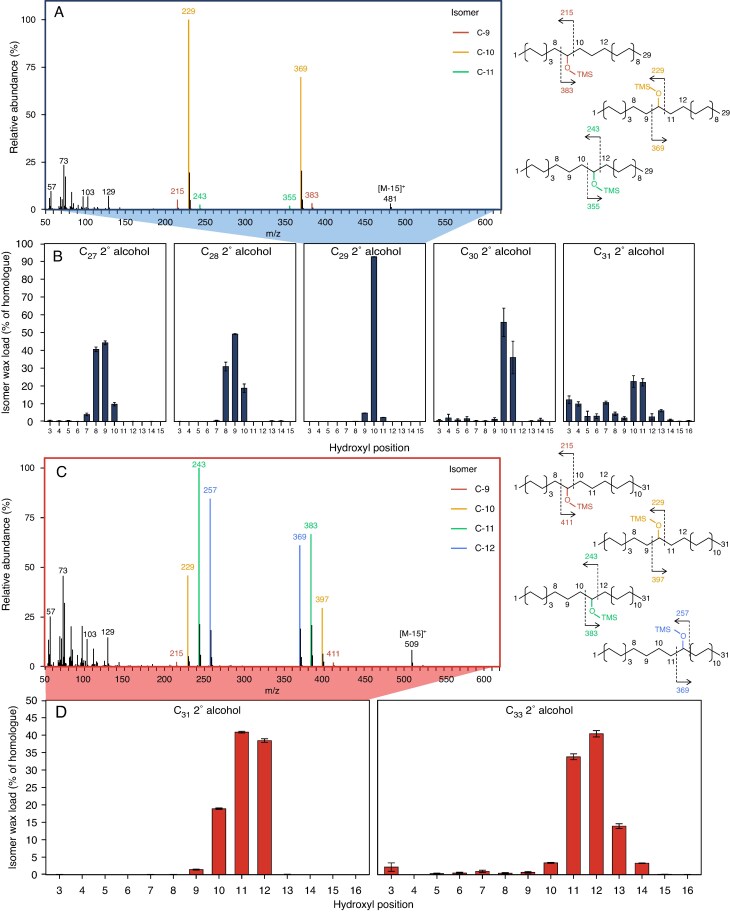
GC–MS identification and isomer profiling of secondary alcohols for select Amygdaloideae and Rosoideae species. (A) Mass spectrum and fragmentation diagram of the *Amelanchier alnifolia* C_29_ secondary alcohol (TMS ether). Characteristic fragment ions of the isomers are highlighted in different colours. (B) Relative isomer amounts for C_27_–C_31_ secondary alcohol homologues of *Amelanchier alnifolia*. (C) Mass spectrum and fragmentation diagram of the *Fragaria chiloensis* C_31_ secondary alcohol (TMS ether). (D) Relative isomer amounts for C_31_ and C_33_ secondary alcohol homologues of *Fragaria chiloensis*. Characteristic fragment ions of the isomers are highlighted in different colours.

To quantify the isomers of each secondary alcohol homologue with variable hydroxyl positions, we performed MS analyses of *Amelanchier alnifolia* and *Fragaria chiloensis* as Amygdaloideae and Rosoideae subfamily representatives, respectively. The mass spectrum of the major secondary alcohol homologue of *Amelanchier alnifolia*, C_29_, displayed combinations of α-fragments *m*/*z* 215/383, *m*/*z* 229/369 and *m*/*z* 243/355, characteristic of isomers with hydroxyls on C-9, C-10 and C-11, respectively (Fig. 5A). Quantitative analyses of respective MS fragments showed that the five *Amelanchier alnifolia* secondary alcohols, with C_27_–C_31_ chain lengths, were dominated by the C-8/-9, C-9, C-10, C-10 and C-10/-11 isomers, respectively (Fig. 5B). In contrast, the dominant C_31_ secondary alcohol of *Fragaria chiloensis* had MS α-fragments indicating the presence of C-9 to C-12 isomers (Fig. 5C). Quantitation of respective fragments revealed that the C_31_ and C_33_ secondary alcohols of *Fragaria chiloensis* were both dominated by the C-11 and C-12 isomers. Thus, hydroxyl groups on even- and odd-numbered carbons were detected for each homologue in both species.

## DISCUSSION

This study compares the micromorphology and composition of cuticular waxes lining the leaves of Rosaceae species. We found that (1) epicuticular wax crystal presence and shapes varied widely, where several Amygdaloideae species had characteristic wax nanotubes while some Rosoideae had elongated, irregular platelet wax crystals, (2) both the total and compound-specific wax loads varied across species, (3) the characteristic secondary alcohols of both subfamilies had hydroxyl groups mainly on C-10/-11/-12, and (4) Amygdaloideae waxes contained more secondary alcohols, while Rosoideae accumulated higher amounts of primary alcohols and alkanes.

### Compounds associated with wax crystals on Rosaceae leaf surfaces

Across the surveyed Rosaceae species, we observed a variety of EWC forms and some distinctions in micromorphology at the subfamily level. Two prominent EWC morphologies, tubules and elongated, irregular platelets, appeared discretely among Amygdaloideae and Rosoideae species, respectively. The wax tubules seen on Amygdaloideae leaf surfaces were well defined, matching earlier reports for closely related species (Neinhuis and Barthlott, 1997; Ganeva and Uzunova, 2010; Tomaszewski and Zieliński, 2014; Song *et al.*, 2024). In contrast, irregular platelets in members of the Rosoideae subfamily were not well defined, though their presence matched literature observations for Rosoideae species (Neinhuis and Barthlott, 1997; Wissemann, 2000; Kim *et al.*, 2009; Tomaszewski and Zieliński, 2014) referring to them as triangular rodlets (Barthlott *et al.*, 1998). This conservation of morphologies within the two subfamilies is confirmed by HCA of our findings of relative wax amounts (Supplementary Data Fig. S9). In particular, it reveals clusters of species of the same subfamily with shared epicuticular wax crystal morphologies (i.e. nanotubes, irregular platelets).

To infer which wax constituents form the EWCs lining Rosaceae leaf surfaces, we compare our wax composition results with previously stated chemistry–structure relationships. Tubular EWCs have been described across diverse lineages in species whose cuticular waxes are dominated by the C_29_ secondary alcohol 10-nonacosanol (Busta and Jetter, 2018). Furthermore, C_29_ diols are commonly found accompanying 10-nonacosanol in wax mixtures (Jetter and Riederer, 1996; Prügel and Lognay, 1996), and have been shown to enhance tubule formation by co-crystallizing with the secondary alcohol (Jetter and Riederer, 1995). Accordingly, the tubule-bearing species investigated here, *Amelanchier alnifolia*, *Spiraea lucida* and *Oemleria cerasiformis*, had wax mixtures rich in 10-nonacosanol and C_29_ diols. This finding is further underlined by PCAs which showed a strong association between the presence of tubular EWCs in Rosaceae species and the accumulation of C_29_ compounds, secondary alcohols and diols in their leaf waxes. Taking these findings together, we conclude that the wax tubules on the leaf surface of various Amygdaloideae result from 10-nonacosanol crystallization.

Though the general tubular aspect of EWCs is preserved between species, we observed slight variations in tubule shape. Tubules in *Amelanchier alnifolia* and *Oemleria cerasiformis* were similar in length but had larger diameters than those of *Spiraea lucida*. These differences may be attributed to the varying amounts of co-crystallizing epicuticular compounds, and likely most importantly, diols (Jetter and Riederer, 1995). Indeed, diols represent 2 % of the total wax mixture in *Amelanchier alnifolia* and *Oemleria cerasiformis* compared with <1 % in *Spiraea lucida*. Future *in vitro* recrystallization experiments of wax mixtures with varying secondary alcohol:diol ratios could provide further insight into the mechanisms causing these subtle morphological differences.

While irregular platelets, such as those found on certain Rosoideae leaves, had previously been qualitatively documented for other families, their chemistry was not discussed in the literature. Here, species bearing this EWC morphology had wax mixtures dominated by C_31_ and C_33_ alkanes, particularly *Fragaria chiloensis* and *Rosa nutkana*. Although still dominant in *Sanguisorba canadensis*, alkanes constituted a smaller proportion of the wax mixture relative to the other irregular platelet-bearing species. In addition, higher amounts of C_31_ and C_33_ secondary alcohols were present in the wax mixtures of these species compared with Rosoideae without irregular platelets. PCAs showed positive associations for platelet-bearing species with the C_31_ and C_33_ chain lengths, alkanes and aldehydes. Based on these observations, we propose that C_31_ and C_33_ alkanes together with secondary alcohols may be involved in forming the irregular platelets in Rosaceae.

Though alkanes are not often discussed as crystal-forming compounds, they are the majority compounds in the wax mixtures of crystal-bearing Brassicaceae. In the stem wax of *Arabidopsis* (Jenks *et al.*, 1995, 2002) and leaf wax of *Brassica oleracea* (Laila *et al.*, 2017), C_29_ alkane is accompanied by notable amounts of crystal-forming secondary alcohols and ketones of the same chain length. The resulting micromorphological phenotypes are a mixture of wax rodlets, platelets and granules. A similar line of reasoning may explain the irregular platelets in Rosaceae. Here, we speculate that the presence, albeit minor, of secondary alcohols alongside alkanes of the same chain length (i.e. C_31_, C_33_) could result in the co-crystallization of these compounds. C_31_ and C_33_ secondary alcohols have not been demonstrated to form distinct wax crystals; however, their similarity in structure to 10-nonacosanol, which forms tubules, is worth noting. The ill-defined nature of this crystal type may thus be explained by two compound classes forming mixed crystals, where alkanes serve as a crystal scaffold allowing smaller amounts of classical crystallizing wax compounds, especially of the same chain length, to form EWCs. While only a hypothesis, it will be interesting to test using *in vitro* recrystallization experiments whether these putative crystal-forming compounds self-assemble as irregular platelets.

Overall, our findings agree with the hypothesis that elevated accumulation of specific compounds results in the formation of EWCs with distinct morphologies (Jetter and Riederer, 1994). In particular, tubules are formed by 10-nonacosanol with admixture of alkanediols, whereas elongated, irregular platelets are speculated to consist primarily of C_31_ and C_33_ alkanes and minor amounts of C_31_ and C_33_ secondary alcohols.

### Requirements for epicuticular wax crystal formation on Rosaceae leaf surfaces

While some of the Rosaceae surveyed here had tubular or platelet-shaped wax crystals on their leaf surfaces, several other species in the Amygdaloideae and Rosoideae subfamilies lacked wax surface microstructures. The occurrence of a smooth layer or crust in these species matches similar reports for the same genera (Tomaszewski and Zieliński, 2014). It remains unclear, however, why closely related species show stark differences in the presence or absence of micromorphology. For example, the crystal-bearing species *Amelanchier alnifolia* and *Fragaria chiloensis* are contrasted by their respective close relatives *Malus fusca* and *Potentilla indica*, which have microscopically smooth leaf surfaces.

To better understand the requirements for EWC formation, we can compare wax traits across the various species investigated here. The amount of wax on the leaf surface is one factor thought to govern the formation of EWCs (Jeffree *et al.*, 1975; Tunstad *et al.*, 2024). The total leaf wax ranged widely (from 3 to 39 µg cm^−2^) across our sampled species, confirming scattered previous reports for Rosaceae (Jetter *et al.*, 2000; Buschhaus *et al.*, 2007; Hunsche and Noga, 2011; Guo *et al.*, 2015; Huang, 2017; Tunstad *et al.*, 2024). Interestingly, our survey revealed that total wax amounts were not a shared trait between closely related species in either subfamily, suggesting that species capacities for wax formation can be tuned to various levels in relatively short evolutionary developments. However, Amygdaloideae species with surface wax tubules and Rosoideae species with irregular platelets had similar high wax loads, in accordance with previous conclusions for other families linking crystal formation to the accumulation of sufficient overall wax amounts (and of specific compounds). In this context, it is interesting to note that some Rosaceae species with intermediate wax loads of ∼20 µg cm^−2^ (*Malus fusca* and *Rubus armeniacus*) and high wax coverage of ∼30 µg cm^−2^ (*Prunus emarginata*) lacked well-defined leaf surface EWCs, whereas *Sanguisorba canadensis* leaves bore wax microstructures at wax loads of ∼15 µg cm^−2^. Together, our findings thus suggest that the formation of EWCs requires the accumulation of critical total wax amounts but also depends on the localization of compounds within the cuticle.

To form EWCs, compounds must accumulate in the epicuticular layer of the cuticle. Accordingly, the dominant compound, 10-nonacosanol, and, more generally, secondary alcohols are known to accumulate in the epicuticular wax fraction of tubule-bearing plant species (Buschhaus *et al.*, 2007). Similarly, the alkanes predicted to make up irregular platelets lining leaf surfaces of some Rosoideae have also been found to accumulate, at least in part, in the epicuticular wax of diverse plants. Therefore, the concentrations of these compounds in respective epicuticular wax compartments can be expected to be equal to or higher than those determined for the Rosaceae total wax mixtures (which include the species’ intracuticular waxes). Thus, our findings agree with previous hypotheses that crystallizing wax components must accumulate to at least 15 % before they can phase-separate from the wax mixture to form EWCs (Gülz *et al.*, 1992).

Conversely, we can explain the lack of distinct EWCs on leaves of species with relatively high total wax loads. Our wax analyses and PCA showed that the *Rubus armeniacus* leaf wax mixture comprised high amounts of alkyl esters. Though esters are known to partially accumulate in the epicuticular fraction of Rosaceae (Buschhaus *et al.*, 2007), this compound class is not associated with the formation of distinct crystal structures (Jeffree *et al.*, 1976). *Malus fusca* and *Prunus emarginata* waxes, on the other hand, comprised high amounts of triterpenoids, which are known to partition in the intracuticular layer (Buschhaus *et al.*, 2007) and would therefore not contribute to EWC formation. This is similarly observed in *Prunus laurocerasus*, whose leaves and fruit have wax loads >60 µg cm^−2^, with wax mixtures primarily consisting of triterpenoids, and lack distinct EWCs (Jetter *et al.*, 2000; Diarte *et al.*, 2021). The aliphatic compounds (e.g. alkanes, primary alcohols) that constitute the rest of its wax mixtures accumulate in insufficient amounts in the epicuticular layer to crystallize. Conversely, in *Vitis vinifera* fruit, primary alcohols are abundant enough to crystallize as wax platelets, despite triterpenoids being the dominant compound class (Arand *et al.*, 2021). Exceptionally, *Macaranga* species bear thread-like surface crystals formed by wax triterpenoids (Markstädter *et al.*, 2000), likely because aliphatic compounds that could interfere with triterpenoid crystallization are absent. Therefore, in the case of Rosaceae, we propose that certain wax-rich species lack EWCs because of a deficiency of aliphatic compounds in the epicuticular layer.

### Biosynthesis of wax secondary alcohols in Rosaceae

The formation of characteristic EWCs on Rosoideae and Amygdaloideae leaf surfaces depends in large part on the accumulation and structure of secondary alcohols in wax mixtures. The mechanisms by which these wax components are formed in Rosaceae can be interpreted from our chemical analyses, in particular the chain length and isomer distributions of relevant compound classes.

The patterns of secondary alcohol isomers discriminate between two biosynthesis mechanisms described for other plant families. The first mechanism involves mid-chain hydroxylation of preformed alkanes, as exemplified by the *Arabidopsis* P450-dependent enzyme MAH1, which hydroxylates C_29_ alkane primarily at the C-15 position to form 15-nonacosanol, but also at adjacent carbons to form 12-, 13- and 14-nonacosanol as by-products (Greer *et al.*, 2007; Wen and Jetter, 2009). Thus, the alkane hydroxylation mechanism yields mixtures of secondary alcohol isomers with hydroxyls on even- and odd-numbered carbons. In contrast, the second mechanism involves the Claisen condensation of two acyl intermediates of wax biosynthesis, leaving hydroxyl groups exclusively on even-numbered carbons (Busta and Jetter, 2018). Here, we found that the secondary alcohols accumulating in various Rosaceae leaf waxes have hydroxyls primarily on even-numbered carbons (e.g. in 10-nonacosanol) but also on adjacent sites (e.g. 9-nonacosanol, 11-nonacosanol) and, thus, at even- and odd-numbered carbons. Therefore, we conclude that the Rosaceae secondary alcohols are biosynthesized through hydroxylation of preformed wax alkanes, similar to *Arabidopsis* but with hydroxyls at/near C-10 instead of C-15. It is plausible that the Rosaceae employ P450-dependent enzymes for secondary alcohol formation like the *Arabidopsis* enzyme MAH1.

The chain-length distributions of alkane pathway products may help predict the substrate specificities of the putative MAH1-like enzymes forming secondary alcohols in the Amygdaloideae and Rosoideae analysed here. Comparisons of the homologue patterns between the predicted alkane substrates and the resulting secondary alcohol products reveal three different patterns across the surveyed Rosaceae: (1) many species in both subfamilies had secondary alcohols dominated by 10-nonacosanol along with C_29_ as the predominant alkane homologue, suggesting that overall product specificity is established during formation of the alkane precursor. However, it cannot be ruled out that further chain length preference is imposed by the alkane-hydroxylating enzyme forming the secondary alcohols. (2) Alkanes in *Spiraea lucida* leaf wax were dominated by the C_31_ homologue, whereas the accompanying secondary alcohols comprised mainly C_29_ chains, implying a strong preference of the hydroxylating enzyme for the C_29_ alkane substrate. (3) Rosoideae species that accumulated C_31_ and C_33_ secondary alcohols also accumulated high quantities of C_31_ and C_33_ alkanes, the hypothesized hydroxylation substrates. However, C_31_ secondary alcohols were more prominent than respective C_33_ homologues in *Fragaria chiloensis* and *Sanguisorba canadensis* leaf waxes, despite C_33_ alkane being more abundant than C_31_ alkane. Thus, our results suggest the presence of an MAH1-like enzyme preferring C_31_ alkane substrate over its C_33_ homologue. Overall, the putative hydroxylating enzymes forming wax secondary alcohols in various Rosaceae appear to have characteristic substrate preferences, with subtle differences between subfamilies and species.

**Fig. 6.**
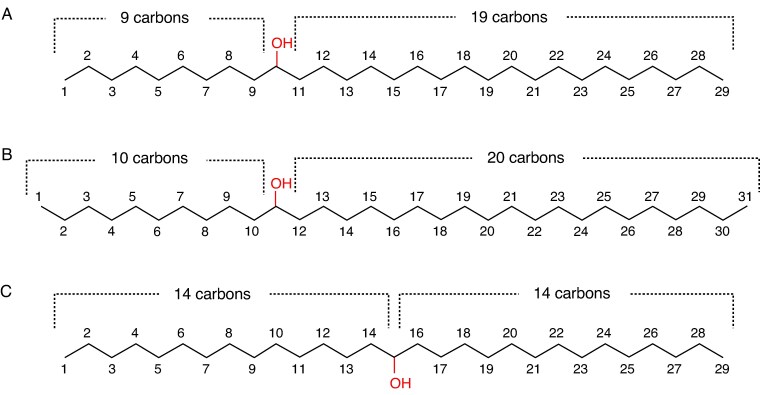
Representative symmetric and asymmetric wax secondary alcohols. Asymmetric (A) 10-nonacosanol and (B) 11-hentriacontanol as found in Rosaceae. (C) Symmetric 15-nonacosanol as found in *Arabidopsis thaliana*.

Finally, our analyses of secondary alcohol isomer profiles help assess the product specificities of the predicted hydroxylating enzymes. All the Rosaceae surveyed had asymmetric secondary alcohols with hydroxyl groups located one-third of the way along the carbon chain (Fig. 6A, B), resulting in a characteristic 1:2 ratio of alkyl chain lengths on either side of the functional group. In contrast, the *Arabidopsis* wax secondary alcohols are symmetric and hydroxylated at/near the centre of the carbon chain (Fig. 6C). Currently, the differences between *Arabidopsis* MAH1 and the Rosaceae hydroxylating enzymes that cause this divergence in product specificities are unclear. However, the characteristic asymmetry of Rosaceae secondary alcohols resulting from specific hydroxylation at/near C-10 may have functional significance for the formation of wax tubules (Jetter and Riederer, 1994).

### Relative contributions of parallel pathways to wax biosynthesis in Rosaceae

The differences in secondary alcohol formation between various Rosaceae must be further considered in the context of overall wax biosynthetic fluxes in the Rosoideae and Amygdaloideae. To this end, the wax profiles reported here can be interpreted in light of current knowledge of wax biosynthesis, particularly by comparing the accumulation of the alcohol-forming and alkane-forming pathway products.

In the alcohol-forming pathway, even-numbered VLCFAs are reduced to even-numbered VLC primary alcohols. Our wax surveys showed that primary alcohols tended to be more abundant in Rosoideae than in Amygdaloideae waxes, and that both subfamilies had primary alcohol average chain lengths consistent with the previously established preference for C_26_ and C_28_ primary alcohols in other families (Baker, 1982; Gülz *et al.*, 1992). Downstream in this pathway, primary alcohols are condensed with long-chain fatty acids to form wax alkyl esters. Accordingly, we found esters more abundant in Rosoideae than in Amygdaloideae wax, while across subfamilies ester average chain lengths were consistent with a general preference for intermediate chain lengths reported for other taxa (Buschhaus *et al.*, 2007; Diarte *et al.*, 2021). Overall, our observations are also in agreement with scattered previous reports showing that, on average, Rosoideae species (Shepherd *et al.*, 1999; Guo *et al.*, 2015; Cheng *et al.*, 2019; Jiang *et al.*, 2024) accumulate more alkyl esters and primary alcohols in their cuticular waxes than Amygdaloideae species (Jetter *et al.*, 2000; Huang, 2017). Therefore, all existing evidence suggests that Rosoideae tend to funnel higher portions of their VLC acyl-CoA precursors into the alcohol-forming pathway relative to the Amygdaloideae.

In the alkane-forming pathway, primarily even-numbered VLC fatty acyl-CoAs are first reduced to corresponding aldehydes, which are then decarbonylated into alkanes with one less carbon (Samuels *et al.*, 2008). Our wax analyses showed aldehydes to be a minor component of Rosaceae leaf wax mixtures, and PCA supported their biosynthetic relationship with the accompanying, primarily odd-numbered alkanes. These alkanes constituted, on average, a greater portion of the Rosoideae leaf wax mixtures than those of Amygdaloideae. This aligns with previous reports, where alkanes dominated the wax compositions of related Rosoideae species (Buschhaus *et al.*, 2007; Guo *et al.*, 2015; Jiang *et al.*, 2024). Taken together, our findings suggest relatively strong flux through the alkane-forming pathway in Rosaceae, with a preference for alkane formation over aldehyde export.

The alkanes formed by aldehyde decarbonylation may serve as substrates for hydroxylation by MAH1-like enzymes, producing secondary alcohols with odd carbon numbers (Greer *et al.*, 2007; Wen and Jetter, 2009). Accordingly, mostly odd-numbered secondary alcohol homologues were detected here, confirming earlier reports on wax mixtures of related Amygdaloideae (Ismail *et al.*, 1976; Knowles *et al.*, 1996; Huang, 2017) and Rosoideae species (Baker and Hunt, 1979; Buschhaus *et al.*, 2007). We further found that Rosaceae leaf waxes with elevated amounts of 10-nonacosanol tended to also accumulate alkanediols, which had previously been recognized as biosynthetic derivatives of the secondary alcohol (Jetter and Riederer, 1995; Prügel and Lognay, 1996; Wen *et al.*, 2006). Thus, the alkane-forming pathway of several Rosaceae encompasses hydroxylation of the alkanes to secondary alcohols and further hydroxylation to diols.

Our comparative analyses revealed that secondary alcohols (and diols) were more abundant in the leaf waxes of Amygdaloideae than those of Rosoideae, and represented the predominant compound class in *Amelanchier alnifolia*, *Spiraea lucida* and *Oemleria cerasiformis* wax mixtures. The *Amelanchier alnifolia*, *Spiraea lucida* and *Oemleria cerasiformis* waxes had greater portions of secondary alcohols relative to alkanes, demonstrating a metabolic preference for converting alkanes to secondary alcohols over directly exporting the alkanes to the cuticle. The opposite is true in Rosoideae, where the higher alkane and, to an extent, aldehyde loads, relative to secondary alcohols, indicate reduced activity or absence of hydroxylating enzymes, and thus prioritization of alkane export. PCA further highlighted this opposing relationship, with Amygdaloideae having a positive association with secondary alcohols and Rosoideae a negative one.

It may be speculated that the formation of secondary alcohols in Amygdaloideae is driven by the functional importance of the nanotubes formed by 10-nonacosanol on their leaf surfaces. Such crystals are known to greatly enhance surface hydrophobicity, thus affecting plant–insect and plant–pathogen interactions as well as UV reflectance (Neinhuis and Barthlott, 1997; Bargel *et al.*, 2006; Middleton *et al.*, 2024; Nobusawa *et al.*, 2025). Conversely, the reduced hydroxylation enzyme activity in Rosoideae, and the ensuing loss of secondary alcohol accumulation and epicuticular wax tubules, may reflect further rapid functional modification of the cuticle over evolutionary time. Currently, the underlying selective force for this remains unclear, considering the potential eco-physiological drawbacks to losing tubules. Because they are poorly characterized in the literature, it is unknown whether the physiological role of irregular platelets is comparable to that of nanotubes.

### Conclusions

Our findings expand the concept within Rosaceae that crystal morphology largely depends on the nature and sufficient accumulation of crystallizing compounds in the wax mixture. Specifically, we show that characteristic wax nanotubes are formed primarily by 10-nonacosanol and propose that jagged and irregular platelets are formed by alkanes co-crystallizing with small amounts of secondary alcohols of similar chain length.

Secondly, our chemical analyses revealed a large variation of wax compositions within subfamilies of Rosaceae, showing that relative fluxes through parallel pathways as well as through individual pathway steps differ between genera. Most notably, homologue and isomer profiles suggest that wax secondary alcohols of Rosaceae are formed by hydroxylation of alkanes, likely catalysed by P450-dependent enzymes. Respective Amygdaloideae and Rosoideae hydroxylases vary in activity, substrate chain length specificities and product regio-specificities, yet produce secondary alcohols with a conserved, 1:2 ratio of alkyl chain lengths on both sides of the hydroxyl group (e.g. 10-nonacosanol, 11-hentriacontanol). This chain length ratio has been hypothesized to be important for tubule formation (Jetter and Riederer, 1994).

Differences in crystal morphology and general wax biosynthetic pathway preferences between species mirror the evolutionary history of Rosaceae subfamilies. Modifications in wax pathway preferences, as evidenced by different ratios of primary alcohol and alkane wax products, and resulting drastic changes in prominent EWC forms, appear to have evolved relatively quickly over the 100 million years since Amygdaloideae and Rosoideae diverged. Seemingly subfamily-specific pathway fluxes and EWC morphologies, however, are lost at lower taxonomic ranks (e.g. tribes). This is demonstrated by the HCA of our findings where, although perfect subfamily clusters do not emerge, the dendrogram largely discriminates between species in each subfamily with and without prevalent epicuticular wax structures (Supplementary Data Fig. S9). This illustrates the relatively quick rate at which biosynthetic fluxes and preferences may shift, resulting in drastically different EWCs within one family, as exemplified by Rosaceae. To what extent these changes reflect specific functional optimizations of the cuticle remains unresolved.

## Supplementary Material

mcaf308_Supplementary_Data

## SUPPLEMENTARY DATA

Supplementary data are available at *Annals of Botany* online and consist of the following. Table S1: Extracted leaf surface area and total wax load. Table S2: Ribulose-1,5-bisphosphate carboxylase/oxygenase large subunit (*rbcL*) gene partial coding sequences. Table S3: Relative loads of wax compound classes. Table S4: Relative loads of individual wax compounds in Amygdaloideae. Table S5: Relative loads of individual wax compounds in Rosoideae. Table S6: Relative loads of pooled aliphatic wax compound chain lengths in Amygdaloideae. Table S7: Relative loads of pooled aliphatic wax compound chain lengths in Rosoideae. Table S8: Average chain length of aliphatic wax compounds across species. Figure S1: Scanning electron micrographs of adaxial and abaxial leaf surfaces for supplementary Rosaceae. Figure S2: Relative loads of wax compound classes for supplementary Rosaceae. Figure S3: Homologue distribution of wax compound classes for main Amygdaloideae. Figure S4: Homologue distribution of wax compound classes for supplementary Amygdaloideae. Figure S5: Homologue distribution of wax compound classes for main Rosoideae. Figure S6: Homologue distribution of wax compound classes for supplementary Rosoideae. Figure S7: GC–MS identification of nonacosane-5,10-diol. Figure S8: Relative loads of pooled aliphatic wax compound chain lengths for supplementary Rosaceae. Figure S9: Agglomerative hierarchical clustering analysis (HCA) of Rosaceae based on relative wax compositional data.
