# Supplementary material for: Composition and structure of Rosaceae leaf cuticles: insights into crystal formation and secondary alcohol biosynthesis
Source: Ann Bot. 2025 Dec 2;137(4):961–74. doi: 10.1093/aob/mcaf308 (PMC13095875; doi:10.1093/aob/mcaf308)
Supplement: mcaf308_Supplementary_Data [file mcaf308_supplementary_data.pdf]

Supplementary Data

Composition and structure of Rosaceae leaf cuticles: Insights into crystal formation and secondary alcohol biosynthesis

The following supplementary tables and figures are included in this PDF:

Table S1: Extracted leaf surface area and total wax load. **Table S2**: Ribulose-1,5-bisphosphate carboxylase/oxygenase large subunit (rbcL) gene partial coding sequences. **Fig. S1**: Scanning electron micrographs of adaxial and abaxial leaf surfaces for supplemental Rosaceae. **Table S3**: Relative loads of wax compound classes. **Table S4**: Relative loads of individual wax compounds in Amygdaloideae. **Table S5**: Relative loads of individual wax compounds in Rosoideae. **Fig. S2**: Relative loads of wax compound classes for supplemental Rosaceae. **Table S6**: Relative loads of pooled aliphatic wax compound chain lengths in Amygdaloideae. **Table S7**: Relative loads of pooled aliphatic wax compound chain lengths in Rosoideae. **Table S8**: Average chain length of aliphatic wax compounds across species. **Fig. S3**: Homologue distribution of wax compound classes for main Amygdaloideae. **Fig. S4**: Homologue distribution of wax compound classes for supplemental Amygdaloideae. **Fig. S5**: Homologue distribution of wax compound classes for main Rosoideae. **Fig. S6**: Homologue distribution of wax compound classes for supplemental Rosoideae. **Fig. S7**: GC-MS identification of nonacosane-5,10-diol. **Fig. S8**: Relative loads of pooled aliphatic wax compound chain lengths for supplemental Rosaceae. **Fig. S9**: Agglomerative hierarchical clustering analysis (HCA) of Rosaceae based on relative wax compositional data.

Table S1 Extracted leaf surface area (cm^2) and total wax load ($\mu g\ cm^{-2}$) per species. Values are the means of biological replicates (n = 5) with error represented as standard error.

Species	Surface area (cm^2)	Wax load ($\mu g\ cm^{-2}$)
M. fusca	26.10 ± 5.85	18.83 ± 2.97
A. alnifolia	21.54 ± 5.85	39.07 ± 4.69
O. cerasiformis	42.76 ± 12.02	37.31 ± 5.9
P. emarginata	22.35 ± 6.88	29.88 ± 7.36
S. lucida	34.59 ± 7.57	27.45 ± 2.53
A. dioicus	45.55 ± 13.79	3.88 ± 0.43
S. canadensis	82.01 ± 11.96	13.13 ± 0.68
P. indica	31.15 ± 8.49	2.95 ± 0.71
F. chiloensis	41.71 ± 14.16	32.17 ± 2.29
R. nutkana	29.77 ± 15.77	36.50 ± 2.63
R. armeniacus	87.69 ± 31.53	18.82 ± 3.8
R. spectabilis	81.21 ± 14.81	3.98 ± 0.4

Table S2 Partial coding sequences of ribulose-1,5-bisphosphate carboxylase/oxygenase large subunit (rbcL) genes from NCBI Nucleotide database (552 bp), in FASTA format, used for multiple sequence alignment (MSA).

Species	Sequence
Malus fusca	>MG249134.1<br>AGTGTTGGATTCAAAGCTGGTGTTAAAGATTATAAATTGACTTATTATACTCCTGACTATGAAACCAAAG<br>ATACTGATATTTTGGCAGCATTTCGAGTAACTCCTCAACCTGGAGTTCCACCTGAGGAAGCAGGGGCCGC<br>GGTAGCTGCTGAATCTTCTACTGGTACATGGACAACTGTATGGACTGACGGTCTTACCAGTCTTGATCGT<br>TACAAAGGTCGATGCTACCACATCGAGCCTGTTGCTGGAGAAGAAAGTCAATTTATTGCTTATGTAGCTT<br>ACCCCTTAGACCTTTTTGAAGAAGGTTCTGTTACTAACATGTTTACTTCCATTGTAGGTAATGTGTTTGG<br>GTTCAAGGCCCTGCGCGCTCTACGTCTGGAGGATTTGCGAATCCCTGTTGCTTATGTTAAAACTTTCCAG<br>GGCCCGCCTCATGGTATCCAAGTTGAGAGAGATAAATTGAACAAGTATGGCCGCCCTCTATTGGGATGTA<br>CTATAAAACCAAAATTGGGGTTATCCGCTAAGAATTACGGTAGAGCAGTTTATGAATGTCTA
Amelanchier alnifolia	>MG248845.1<br>AGTGTTGGATTCAAAGCTGGTGTTAAAGATTATAAATTGACTTATTATACTCCTGACTATGAAACCAAAG<br>ATACTGATATTTTGGCAGCATTTCGAGTAACTCCTCAACCTGGAGTTCCACCTGAGGAAGCAGGGGCCGC<br>GGTAGCTGCTGAATCTTCTACTGGTACATGGACAACTGTATGGACTGACGGTCTTACCAGTCTTGATCGT<br>TACAAAGGTCGATGCTACCACATCGAGCCTGTTGCTGGAGAAGAAAGTCAATTTATTGCTTATGTAGCTT<br>ACCCCTTAGACCTTTTTGAAGAAGGTTCTGTTACTAACATGTTTACTTCCATTGTAGGTAATGTGTTTGG<br>GTTCAAGGCCCTGCGCGCTCTACGTCTGGAGGATTTGCGAATCCCTACTGCTTATGTTAAAACTTTCCAG<br>GGCCCGCCTCATGGTATCCAAGTTGAGAGAGATAAATTGAACAAGTATGGCCGCCCTCTATTGGGATGTA<br>CTATAAAACCAAAATTGGGGTTATCCGCTAAGAATTACGGTAGAGCAGTTTATGAATGTCTA

Species	Sequence
Oemleria cerasiformis	>MG249742.1<br>AGTGTTGGATTCAAAGCTGGTGTTAAAGAGTATAAATTGACTTATTATACTCCTGAATATGAAACCAAAG<br>ATACTGATATCTTGGCAGCATTTCGAGTAACTCCTCAACCTGGAGTTCCACCTGAGGAAGCGGGGGCCGC<br>AGTAGCTGCTGAATCTTCTACTGGTACATGGACAACTGTATGGACTGACGGGCTTACTAGTCTTGATCGT<br>TACAAAGGTCGATGCTACCACATCGAGCCCGTTCCTGGAGAAGAAAGTCAATTTATTGCTTATGTAGCTT<br>ACCCCTTAGACCTTTTTGAAGAAGGTTCTGTTACTAACCTGTTTACTTCCATTGTAGGTAATGTGTTTGG<br>GTTTAAGGCCCTGCGCGCTCTACGTCTGGAGGATTTGCGAATCCCTCCTGCTTATGTTAAAACTTTCCAA<br>GGGCCGCCTCATGGGATCCAAGTTGAGAGAGATAAATTGAACAAGTACGGCCGACCTCTATTGGGATGTA<br>CTATTAAACCTAAATTGGGGTTATCCGCTAAGAATTACGGTAGAGCGGTTTATGAATGTCTC
Prunus emarginata	>MG248675.1<br>AGTGTTGGGTTCAAAGCTGGTGTTAAAGATTATAAATTGACTTATTATACTCCTGACTATGAAACCAAAG<br>ATACTGATATCTTGGCAGCATTTCGAGTAACTCCTCAACCTGGAGTTCCACCTGAAGAAGCAGGGGCAGC<br>GGTAGCTGCTGAATCTTCTACTGGTACATGGACAACTGTATGGACTGACGGGCTTACTAGTCTTGATCGT<br>TACAAAGGTCGATGCTACCACATCGAGCCCGTTGCTGGAGAAGAAAGTCAATTTATTGCTTATGTAGCTT<br>ACCCCTTAGACCTTTTTGAAGAGGGTTCTGTTACTAACATGTTTACTTCCATTGTAGGTAATGTGTTTGG<br>GTTCAAGGCCCTGCGCGCGCTACGTCTGGAGGATTTGCGAATCCCTACTGCTTATGTTAAAACCTTCCAA<br>GGCCCGCCTCATGGGATCCAAGTTGAGAGAGATAAATTGAACAAGTATGGCCGCCCCCTATTGGGATGTA<br>CTATTAAACCTAAATTGGGGTTATCCGCTAAGAATTACGGTAGAGCAGTTTATGAATGTCTC
Spiraea lucida	>MG249884.1<br>AGTGTTGGATTCAAAGCTGGTGTTAAAGAGTATAAATTGACTTATTATACTCCGGACTATGAAACCAAAG<br>ATACTGATATCTTGGCAGCATTTCGAGTAACTCCTCAACCTGGAGTTCCACCTGAGGAAGCAGGGGCCGC<br>GGTAGCTGCTGAATCTTCTACGGGTACATGGACAACTGTATGGACTGACGGGCTTACCAGTCTTGATCGT<br>TACAAAGGTCGATGCTACCACATTGAGCCCGTTGCTGGAGAAGAAAATCAATTTATTGCTTATGTAGCTT<br>ACCCCTTAGACCTTTTTGAAGAAGGTTCTGTTACTAACATGTTTACTTCCATTGTAGGTAATGTGTTTGG<br>GTTCAAGGCCCTGCGTGCTCTACGTCTGGAGGATTTGCGAATCCCTACTGCTTATGTTAAAACTTTCCAA<br>GGCCCGCCTCATGGGATCCAAGTTGAGAGAGATAAATTGAACAAGTATGGACGCCCCCTATTGGGATGTA<br>CTATTAAACCTAAATTGGGGTTATCCGCTAAGAATTACGGTAGAGCAGTTTATGAGTGTCTC
Aruncus dioicus	>MG247899.1<br>AGTGTTGGATTCAAAGCTGGTGTTAAAGATTATAAATTGAATTATTATACTCCTGACTATGAAACCAAAG<br>ATACTGATATCTTGGCAGCATTTCGAGTAACTCCTCAACCTGGAGTTCCACCTGAGGAAGCAGGGGCAGC<br>GGTAGCTGCTGAATCTTCTACGGGTACGTGGACAACTGTATGGACTGACGGGCTTACCAGTCTTGATCGT<br>TACAAAGGTCGATGCTACCACATTGAGCCCGTTGCCGGAGAAGAAAGTCAATTTATTGCTTATGTAGCTT<br>ACCCCTTAGACCTTTTTGAAGAAGGTTCTGTTACTAACATGTTTACTTCCATTGTAGGTAATGTGTTTGG<br>GTTCAAGGCCCTGCGTGCTCTACGTCTGGAGGATTTGCGAATCCCCACTGCTTATGTTAAAACTTTCCAA<br>GGCCCGCCTCACGGGATTCAAGTTGAGAGAGATAAATTGAACAAGTATGGTCGCCCTCTATTGGGATGTA<br>CTATTAAACCTAAATTGGGGTTATCCGCTAAGAATTATGGTAGAGCAGTTTATGAGTGTCTC
Sanguisorba canadensis	>MG249348.1<br>AGTGTTGGATTCAAAGCTGGTGTTAAAGATTATAAATTGACTTATTATACTCCGGACTATGAAACCAAAG<br>ATACTGATATCTTGGCAGCATTTCGAGTAACTCCTCAACCTGGAGTTCCGCCTGAGGAAGCAGGGGCGGC<br>GGTAGCTGCGGAATCTTCTACTGGTACATGGACAACTGTATGGACTGACGGGCTTACCAGTCTTGATCGT<br>TACAAAGGGCGCTGCTACCATATTGAACCTGTTGCTGGAGAAGAAAATCAATTTATTGCTTATGTAGCCT<br>ACCCCTTAGACCTTTTTGAAGAGGGTTCGGTTACTAACATGTTTACTTCCATTGTAGGTAATGTATTTGG<br>GTTCAAGGCCTTGCGCGCTCTACGTCTGGAGGATTTACGAATTCCTCCTGCTTATGTTAAAACTTTCCAA<br>GGCCCGCCTCACGGGATCCAAGTTGAAAGAGATAAATTGAACAAGTACGGCCGCCCGCTATTGGGATGCA<br>CTATTAAACCTAAATTGGGGTTATCCGCTAAGAATTACGGTAGAGCAGTTTATGAATGTCTC
Potentilla indica	>MF694761.1<br>AGTGTTGGATTCAAAGCTGGTGTTAAAGATTATAAATTGACTTATTATACTCCGGAGTATGAAACCAAAG<br>ATACTGATATATTGGCAGCATTTCGAGTAACTCCTCAACCTGGAGTTCCGCCCGAGGAAGCAGGGGCAGC<br>GGTAGCTGCGGAATCTTCTACTGGTACATGGACAACTGTATGGACTGACGGGCTTACCAGTCTTGATCGT<br>TACAAAGGACGATGCTACGGAATTGAACCTGTTCCTGGAGAAGAAAGTCAATTTATTGCTTATGTAGCTT<br>ACCCATTAGACCTTTTTGAAGAGGGTTCGGTTACTAACATGTTTACTTCCATTGTAGGTAATGTGTTTGG<br>GTTCAAGGCCTTGCGTGCTCTACGTCTGGAGGATTTACGAATCCCTACTGCTTATGTTAAAACTTTCCAA<br>GGCCCGCCTCACGGGATCCAAGTTGAAAGAGATAAATTGAACAAGTATGGACGCCCCCTATTGGGATGTA<br>CTATTAAACCTAAATTGGGGTTATCCGCTAAGAATTACGGTAGAGCAGTTTATGAATGTCTT

Species	Sequence
Fragaria chiloensis	>MG246685.1<br>AGTGTTGGATTCAAAGCTGGTGTTAAAGATTATAAATTGACTTATTATACTCCGGACTATGAAACCAAAG<br>ATACTGATATATTGGCAGCATTTCGAGTAACTCCTCAACCTGGAGTTCCGCCTGAGGAAGCAGGGGCAGC<br>GGTAGCTGCGGAATCTTCTACTGGTACATGGACAACTGTATGGACTGACGGGCTTACCAGTCTTGATCGT<br>TACAAAGGGCGATGCTACCACATCGAACCTGTTCCTGGAGAAGAAAGTCAATTTATTGCTTATGTAGCTT<br>ACCCCTTAGACCTTTTTGAAGAGGGTTCGGTTACTAACATGTTTACTTCGATTGTAGGTAATGTGTTTGG<br>GTTCAAGGCCTTGCGCGCTCTACGTCTGGAGGATTTACGAATCCCTACTGCTTATGTTAAAACTTTCCAA<br>GGCCCGCCTCACGGGATCCAAGTTGAAAGAGATAAATTGAACAAGTATGGCCGCCCCCTATTGGGATGTA<br>CTATTAAACCTAAATTGGGGTTATCCGCTAAGAATTACGGTAGAGCAGTTTATGAATGTCTC
Rosa nutkana	>MG248521.1<br>AGTGTTGGATTCAAAGCTGGTGTTAAAGATTATAAATTGACTTATTATACTCCGGATTATGAAACCAAAG<br>ATACTGATATCTTGGCAGCATTTCGAGTAACTCCTCAACCTGGAGTTCCGCCTGAGGAAGCAGGGGCAGC<br>GGTAGCTGCGGAATCTTCTACTGGTACATGGACAACTGTATGGACTGATGGGCTTACCAGTCTTGATCGT<br>TACAAAGGGCGATGCTACCACATTGAACCTGTTGCTGGAGAAGAAAGTCAATTTATTGCTTATGTAGCTT<br>ACCCCTTAGACCTTTTTGAAGAGGGTTCGGTTACTAACATGTTTACTTCCATTGTAGGTAATGTGTTTGG<br>GTTCAAGGCCTTGCGCGCTCTACGTCTGGAGGATTTACGAATCCCTACTGCTTATGTTAAAACTTTCCAA<br>GGCCCGCCTCACGGGATCCAAGTTGAAAGAGATAAATTGAACAAGTATGGCCGCCCCCTATTGGGATGTA<br>CTATTAAACCTAAATTGGGGTTATCCGCTAAGAATTACGGTAGAGCAGTTTATGAATGTCTC
Rubus armeniacus	>KX678957.1<br>AGTGTTGGATTCAAAGCTGGTGTTAAAGATTATAAATTGACTTATTACACTCCAGACTATGAAACCAAAG<br>ATACTGATATCTTGGCAGCATTTCGAGTAACTCCTCAACCTGGAGTTCCGCCTGAGGAAGCAGGGGCCGC<br>GGTAGCTGCGGAATCTTCTACCGGTACATGGACAACTGTATGGACTGACGGGCTTACTAGTCTTGATCGT<br>TACAAAGGGCGATGCTACCACATTGAACCTGTTGCTGGAGAAGAAAGTCAATTTATTGCTTATGTAGCTT<br>ACCCCTTAGACCTTTTTGAAGAAGGTTCGGTTACTAACATGTTTACTTCCATTGTAGGTAATGTGTTTGG<br>GTTCAAGGCCTTGCGCGCTCTACGTCTGGAGGATTTACGAATCCCTCCTGCTTATGTTAAAACTTTCCAA<br>GGCCCGCCTCACGGGATCCAAGTTGAAAGAGATAAATTGAACAAGTATGGCCGCCCCCTATTGGGATGTA<br>CTATTAAACCTAAATTGGGGTTATCCGCTAAGAATTACGGTAGAGCAGTTTATGAATGTCTC
Rubus spectabilis	>KX678946.1<br>AGTGTTGGATTCAAAGCTGGTGTTAAAGAGTATAAATTGACTTATTATACTCCGGACTATGAAACCAAAG<br>ATACTGATATCTTGGCAGCATTTCGAGTAACTCCTCAGCCTGGAGTTCCGCCTGAGGAAGCAGGGGCCGC<br>GGTAGCTGCGGAATCTTCTACCGGTACATGGACAACTGTATGGACTGACGGGCTTACTAGTCTTGATCGT<br>TACAAAGGGCGATGCTACCACATTGAACCTGTTGCTGGAGAAGAAAGTCAATTTATTGCTTATGTAGCTT<br>ACCCCTTAGACCTTTTTGAAGAAGGTTCGGTTACTAACATGTTTACTTCCATTGTAGGTAATGTGTTTGG<br>GTTCAAGGCCTTGCGCGCTCTACGTCTGGAGGATTTACGAATCCCTCCTGCTTATGTTAAAACTTTCCAA<br>GGCCCGCCTCACGGGATCCAAGTTGAAAGAGATAAATTGAACAAGTATGGCCGCCCCCTATTGGGATGTA<br>CTATTAAACCTAAATTGGGGTTATCCGCTAAGAATTACGGTAGAGCAGTTTATGAATGTCTC
***Arbutus menziesii* (outgroup)**	>KX678427.1<br>AGTGTTGGATTCAAAGCTGGTGTTAAAGATTACAAATTGACTTATTATACTCCTAAATATGAAACAAAAG<br>ATACTGATATCTTGGCAGCATTCCGAGTAACTCCTCAACCAGGAGTTCCACCTGAAGAAGCAGGGGCCGC<br>GGTAGCTGCAGAATCTTCTACTGGTACATGGACAACTGTGTGGACCGATGGACTTACTAGCCTTGATCGT<br>TACAAAGGGCGATGCTACCACATCGAGCCTGTTGCTGGAGAAGAAAATCAATATATTGCTTATGTAGCTT<br>ATCCTTTAGACCTTTTTGAAGAAGGTTCCGTTACTAACATGTTTACTTCCATTGTGGGTAATGTATTTGG<br>GTTCAAAGCCCTGCGCGCTCTACGTCTGGAAGATCTACGAATCCCTGTTGCGTATGTTAAAACTTTCCAA<br>GGGCCGCCTCATGGCATCCAAGTTGAAAGAGATAAATTGAACAAGTATGGTCGTCCCCTGTTGGGATGTA<br>CTATTAAACCAAAATTGGGGTTATCTGCTAAAAACTATGGGCGAGCAGTTTATGAATGTCTC

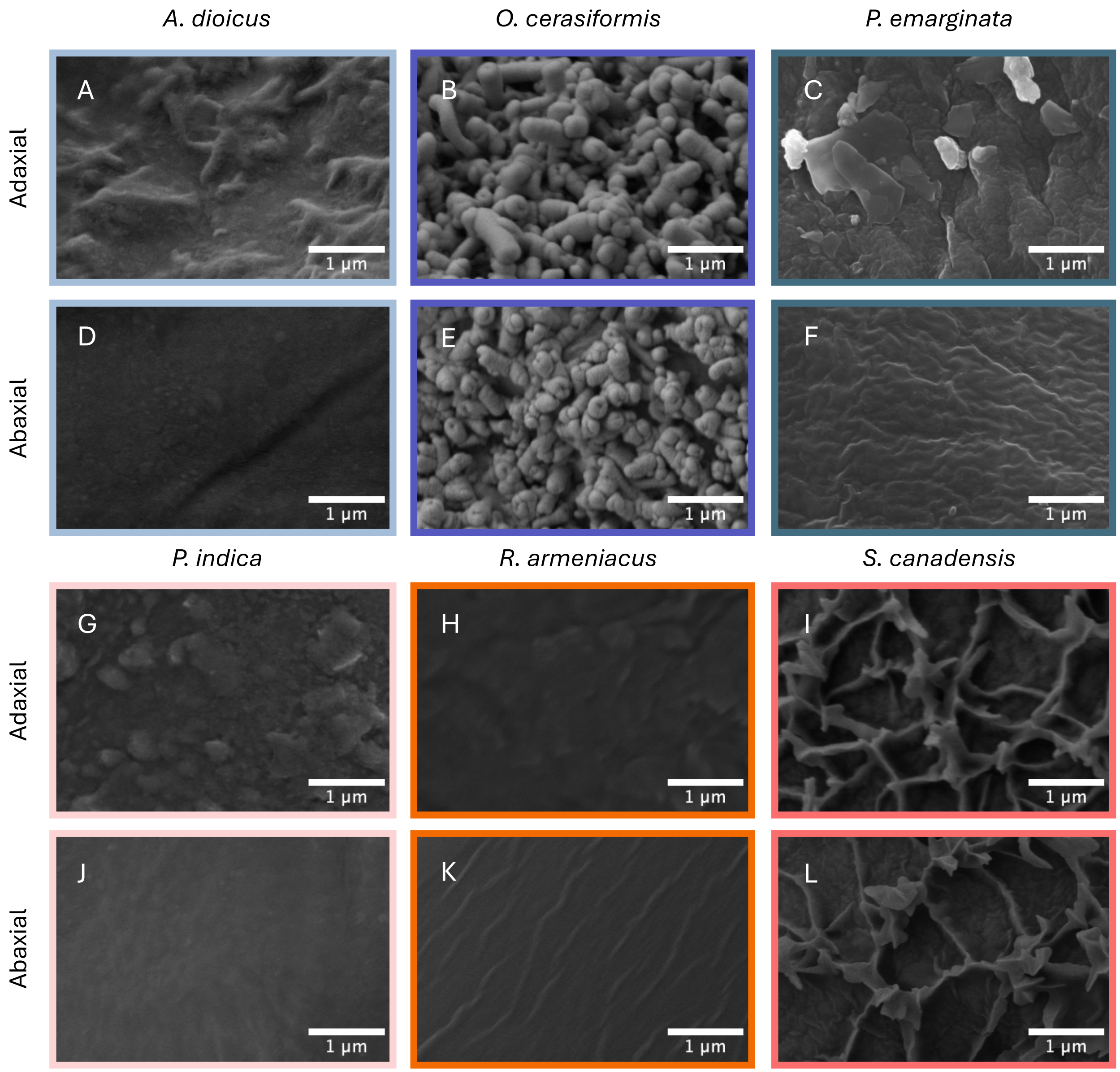

Fig. S1 Scanning electron micrographs of six Rosaceae species' leaf surfaces at high magnification. Adaxial leaf surfaces of Amygdaloideae (A-C) and Rosoideae (G-I) species. Abaxial leaf surfaces of Amygdaloideae (D-F) and Rosoideae species (J-L).

Table S3 Relative loads of individual compound classes. Values are the means of biological replicates (n = 5) with error represented as standard error.

Species	Aldehydes	Alkanes	Secondary alcohols	Diols	Acids	Primary alcohols	Esters	Triterpenoids	Unknowns
M. fusca	2.45 ± 0.17	13.19 ± 1.03	5.15 ± 0.2	0	1.08 ± 0.09	13.7 ± 0.74	11.62 ± 1.24	40.3 ± 3.35	13.58 ± 0.84
A. alnifolia	1.81 ± 0.26	13.14 ± 0.38	54.05 ± 2.28	1.51 ± 0.14	1.26 ± 0.09	3.68 ± 0.27	11.96 ± 1.61	9.81 ± 0.88	4.04 ± 1.14
O. cerasiformis	1.53 ± 0.17	9.05 ± 0.68	30.55 ± 7.19	2.33 ± 0.21	0.85 ± 0.1	6.34 ± 0.66	8.8 ± 0.85	28.22 ± 5.89	13.18 ± 7.78
P. emarginata	0.55 ± 0.2	20.57 ± 1.54	1.21 ± 0.17	0	0.93 ± 0.25	4.73 ± 0.5	7.01 ± 0.52	54.11 ± 1.75	11.82 ± 1.67
S. lucida	1.8 ± 0.14	8.07 ± 0.51	39.88 ± 2.67	0.1 ± 0.02	0.67 ± 0.04	8.15 ± 0.66	28.52 ± 0.85	4.93 ± 0.73	8.55 ± 0.92
A. dioicus	3.17 ± 0.42	10.28 ± 1.41	9.21 ± 1.43	0	0.47 ± 0.09	27.81 ± 1.54	22.46 ± 2.71	10.99 ± 0.53	16.09 ± 1.96
S. canadensis	4.66 ± 0.75	27.53 ± 0.42	2.47 ± 0.33	0	0.48 ± 0.05	15.32 ± 0.5	4.8 ± 0.25	25.54 ± 1.15	19.69 ± 0.63
P. indica	2.15 ± 0.4	67.61 ± 1.5	0.41 ± 0.13	0	0.08 ± 0.02	3.78 ± 0.4	5.6 ± 0.65	3.4 ± 0.3	17.05 ± 0.61
F. chiloensis	2.43 ± 0.45	42.1 ± 1.57	3.15 ± 0.38	0	1.95 ± 0.43	7.7 ± 1.41	20.53 ± 0.55	5.47 ± 0.19	18.62 ± 0.75
R. nutkana	6.53 ± 0.82	39.37 ± 1.02	6.1 ± 0.62	0	1.75 ± 0.11	19.6 ± 1.41	10.64 ± 0.95	1.78 ± 0.2	15.98 ± 0.89
R. armeniacus	1.65 ± 0.3	12.35 ± 1.34	0.29 ± 0.08	0	0.36 ± 0.06	14.2 ± 1.91	42.06 ± 2.42	6.62 ± 0.65	22.82 ± 1.27
R. spectabilis	2.53 ± 0.39	6.76 ± 1.52	0.86 ± 0.32	1.1 ± 0.18	0.17 ± 0.01	44.4 ± 5.01	16.29 ± 2.14	6.43 ± 0.4	21.62 ± 2.31

Table S4 Absolute loads of individual wax compounds in six Amygdaloideae species. Values are the means of biological replicates (n = 5) with error represented as standard error.

Compound	*M. fusca*	*A. alnifolia*	*O. cerasiformis*	*P. emarginata*	*S. lucida*	*A. dioicus*
**Aldehydes**						
**24**	0.022 ± 0	0	0.018 ± 0	0.005 ± 0	0.005 ± 0	0.007 ± 0
**26**	0.06 ± 0.02	0.029 ± 0	0.032 ± 0	0.019 ± 0	0.003 ± 0	0.016 ± 0
**28**	0.109 ± 0.01	0.081 ± 0.01	0.112 ± 0.01	0.004 ± 0	0.02 ± 0.01	0.018 ± 0
**30**	0.23 ± 0.03	0.555 ± 0.07	0.363 ± 0.04	0.124 ± 0.05	0.443 ± 0.02	0.066 ± 0.01
**Alkanes**						
**25**	0.029 ± 0.01	0.03 ± 0.01	0.032 ± 0.01	0.094 ± 0.02	0.006 ± 0	0.082 ± 0.03
**26**	0.033 ± 0	0.073 ± 0.01	0.012 ± 0	0.004 ± 0	0.016 ± 0	0.007 ± 0
**27**	0.102 ± 0.02	0.252 ± 0.03	0.106 ± 0.03	0.363 ± 0.06	0.048 ± 0.01	0.039 ± 0.02
**28**	0.155 ± 0.02	0.061 ± 0.01	0.026 ± 0.01	0.048 ± 0.01	0.007 ± 0	0.004 ± 0
**29**	0.486 ± 0.06	3.857 ± 0.56	2.001 ± 0.19	2.258 ± 0.56	0.799 ± 0.1	0.137 ± 0.04
**30**	0.033 ± 0.01	0.025 ± 0	0.009 ± 0	0.125 ± 0.02	0.009 ± 0	0.047 ± 0.01
**31**	1.189 ± 0.15	0.555 ± 0.07	0.982 ± 0.14	2.474 ± 0.61	1.219 ± 0.08	0.015 ± 0
**32**	0.178 ± 0.03	0.012 ± 0	0.021 ± 0	0.028 ± 0.01	0.006 ± 0	0.02 ± 0.01
**33**	0.035 ± 0	0.109 ± 0.03	0	0.456 ± 0.16	0.017 ± 0.01	0
**Secondary alcohols**						
**27**	0.026 ± 0.01	0.432 ± 0.07	0.016 ± 0	0	0.004 ± 0	0.086 ± 0.03
**28**	0.362 ± 0.04	0.101 ± 0.01	0.051 ± 0.01	0	0.026 ± 0.01	0.018 ± 0.01
**29**	0.022 ± 0.01	19.032 ± 2.7	9.138 ± 2.33	0.017 ± 0	9.705 ± 0.92	0.015 ± 0
**30**	0.01 ± 0	0.147 ± 0.03	0.104 ± 0.04	0	0.114 ± 0.02	0.008 ± 0
**31**	0.484 ± 0.12	0.903 ± 0.08	1.679 ± 0.13	0.358 ± 0.14	0.719 ± 0.04	0.202 ± 0.08

Compound	*M. fusca*	*A. alnifolia*	*O. cerasiformis*	*P. emarginata*	*S. lucida*	*A. dioicus*
**Secondary alcohols**						
**33**	0	0	0	0	0	0
**Diols**						
**29 (4, 10)**	0	0	0.285 ± 0.04	0	0	0
**29 (5, 10)**	0	0.559 ± 0.05	0.559 ± 0.1	0	0.029 ± 0.01	0
**Acids**						
**20**	0.15 ± 0.01	0.261 ± 0.03	0.178 ± 0.01	0.123 ± 0.03	0.14 ± 0.01	0.374 ± 0.09
**22**	0.16 ± 0.03	0.064 ± 0.02	0.031 ± 0.01	0.066 ± 0.01	0.038 ± 0	0.013 ± 0
**24**	0.27 ± 0.04	0.091 ± 0.01	0.026 ± 0.01	0.084 ± 0.01	0.085 ± 0.01	0.02 ± 0
**26**	0.031 ± 0.01	0.101 ± 0.01	0.03 ± 0.01	0.085 ± 0.02	0.027 ± 0	0.028 ± 0
**28**	0.381 ± 0.05	0.701 ± 0.06	0.295 ± 0.03	0.51 ± 0.24	0.27 ± 0.04	0.03 ± 0.01
**30**	0.09 ± 0.02	0.043 ± 0.01	0.286 ± 0.07	0.066 ± 0.02	0.113 ± 0.02	0.008 ± 0
**Primary alcohols**						
**21**	0.036 ± 0	0	0	0.005 ± 0.01	0	0
**22**	0.019 ± 0	0.096 ± 0.02	0.121 ± 0.02	0.015 ± 0	0.056 ± 0.01	0.095 ± 0.02
**23**	0.02 ± 0	0	0.003 ± 0	0.049 ± 0.03	0.001 ± 0	0.001 ± 0
**24**	0.249 ± 0.04	0.053 ± 0.01	0.421 ± 0.08	0.091 ± 0.01	0.148 ± 0.03	0.199 ± 0.02
**25**	0.039 ± 0.01	0.017 ± 0	0.015 ± 0	0.028 ± 0.01	0.005 ± 0	0.01 ± 0
**26**	0.711 ± 0.09	0.127 ± 0.04	0.306 ± 0.03	0.562 ± 0.11	0.07 ± 0.01	0.225 ± 0.03
**27**	0.019 ± 0	0.008 ± 0	0.005 ± 0	0	0.004 ± 0	0.004 ± 0
**28**	0.479 ± 0.05	0.625 ± 0.12	0.366 ± 0.04	0.393 ± 0.09	0.251 ± 0.09	0.079 ± 0.01
**29**	0.04 ± 0.01	0.156 ± 0.01	0.126 ± 0.01	0.042 ± 0.01	0.091 ± 0.01	0.009 ± 0
**30**	0.331 ± 0.05	0.091 ± 0.03	0.859 ± 0.16	0.108 ± 0.04	1.592 ± 0.24	0.246 ± 0.05
**32**	0.476 ± 0.19	0.239 ± 0.07	0	0	0.029 ± 0.01	0.062 ± 0.01
**Esters**						
**38**	0.041 ± 0.01	0.743 ± 0.11	0.142 ± 0.01	0.102 ± 0.01	1.176 ± 0.14	0.013 ± 0.01
**40**	0.069 ± 0.02	1.351 ± 0.13	0.9 ± 0.09	0.193 ± 0.04	2.468 ± 0.24	0.03 ± 0.01
**42**	0.294 ± 0.05	0.747 ± 0.13	0.611 ± 0.06	0.326 ± 0.09	1.651 ± 0.21	0.058 ± 0.02
**44**	0.469 ± 0.04	0.741 ± 0.13	0.463 ± 0.12	0.497 ± 0.22	1.134 ± 0.15	0.156 ± 0.03
**46**	0.336 ± 0.04	0.454 ± 0.07	0.662 ± 0.08	0.398 ± 0.12	0.428 ± 0.05	0.226 ± 0.03
**48**	0.362 ± 0.04	0.205 ± 0.03	0.165 ± 0.04	0.328 ± 0.11	0.473 ± 0.04	0.171 ± 0.02
**50**	0.249 ± 0.03	0.061 ± 0.01	0.053 ± 0.01	0.214 ± 0.08	0.239 ± 0.04	0.064 ± 0.01
**52**	0.113 ± 0.01	0.034 ± 0.01	0.024 ± 0.01	0.09 ± 0.04	0.071 ± 0.01	0.034 ± 0.01
**Triterpenoids**						
**Alpha amyrin**	0	0.438 ± 0.11	0	0.077 ± 0.08	0	0.204 ± 0.03
**Beta amyrin**	0	0.749 ± 0.19	0	0.215 ± 0.08	0	0.138 ± 0.02
**Erythrodiol**	0	0.833 ± 0.07	0	0	0	0
**Oleanoic acid**	1.503 ± 0.39	0.451 ± 0.13	1.605 ± 0.46	4.055 ± 1.05	0	0.009 ± 0
**Ursolic acid**	5.62 ± 1.33	0.961 ± 0.26	9.574 ± 3.84	11.19 ± 2.61	0.899 ± 0.16	0.008 ± 0
**Uvaol**	0.382 ± 0.08	0.363 ± 0.07	0.369 ± 0.09	0	0.464 ± 0.16	0.018 ± 0.01
**Unknowns**	2.322 ± 0.26	1.458 ± 0.37	4.127 ± 2.01	3.591 ± 1.17	2.336 ± 0.44	0.558 ± 0.12

Table S5 Absolute loads of individual wax compounds in six Rosoideae species. Values are the means of biological replicates (n = 5) with error represented as standard error.

Compound	*S. canadensis*	*P. indica*	*F. chiloensis*	*R. nutkana*	*R. armeniacus*	*R. spectabilis*
**Aldehydes**						
**24**	0.022 ± 0	0.023 ± 0.01	0.019 ± 0.01	0.063 ± 0.01	0.014 ± 0	0.01 ± 0
**26**	0.223 ± 0.03	0.012 ± 0	0.209 ± 0.04	0.721 ± 0.15	0.02 ± 0	0.037 ± 0.01
**28**	0.273 ± 0.05	0.004 ± 0	0.376 ± 0.11	1.409 ± 0.21	0.095 ± 0.01	0.044 ± 0.01
**30**	0.061 ± 0.01	0.019 ± 0.01	0.134 ± 0.02	0.134 ± 0.02	0.131 ± 0.01	0.006 ± 0
**Alkanes**						
**25**	0.02 ± 0	0.005 ± 0	0.059 ± 0.01	0.224 ± 0.04	0.028 ± 0.01	0.011 ± 0
**26**	0.031 ± 0	0	0.05 ± 0.01	0.042 ± 0.01	0.012 ± 0	0.01 ± 0
**27**	0.05 ± 0	0.048 ± 0.02	0.307 ± 0.01	0.696 ± 0.08	0.052 ± 0.01	0.047 ± 0.02
**28**	0.003 ± 0	0.007 ± 0	0.01 ± 0	0.012 ± 0	0.015 ± 0	0.003 ± 0
**29**	0.034 ± 0.01	0.085 ± 0.02	0.245 ± 0.02	1.063 ± 0.08	0.198 ± 0.06	0.083 ± 0.03
**30**	0.003 ± 0	0.017 ± 0.01	0.397 ± 0.18	0.079 ± 0.02	0.247 ± 0.04	0.032 ± 0.01
**31**	0.694 ± 0.05	0.808 ± 0.22	4.919 ± 0.52	5.535 ± 0.54	0.274 ± 0.08	0.075 ± 0.03
**32**	0.023 ± 0	0.049 ± 0.02	0.226 ± 0.06	0.117 ± 0.03	1.338 ± 0.21	0.002 ± 0
**33**	2.625 ± 0.13	0.946 ± 0.26	6.621 ± 0.8	5.896 ± 0.39	0	0.005 ± 0
**Secondary alcohols**						
**27**	0.014 ± 0	0	0	0	0.003 ± 0	0.014 ± 0.01
**28**	0	0	0	0	0.054 ± 0.03	0
**29**	0	0.007 ± 0	0	0.05 ± 0.02	0.003 ± 0	0.009 ± 0
**30**	0	0	0	0	0	0
**31**	0.27 ± 0.04	0.004 ± 0	0.851 ± 0.18	1.138 ± 0.09	0	0
**33**	0.035 ± 0.02	0	0.114 ± 0.05	0.903 ± 0.11	0	0.008 ± 0
**Diols**						
**29 (4, 10)**	0	0	0	0	0	0
**29 (5, 10)**	0	0	0	0	0	0.003 ± 0
**Acids**						
**20**	0.004 ± 0	0	0.212 ± 0.06	0.138 ± 0.01	0.115 ± 0.01	0.056 ± 0
**22**	0.006 ± 0	0.011 ± 0	0.151 ± 0.06	0.097 ± 0.02	0.028 ± 0.01	0.013 ± 0
**24**	0.09 ± 0.01	0.007 ± 0	0.301 ± 0.07	0.186 ± 0.02	0.038 ± 0.01	0.012 ± 0
**26**	0.17 ± 0.01	0.007 ± 0	0.186 ± 0.02	0.515 ± 0.08	0.05 ± 0.02	0.021 ± 0.01
**28**	0.167 ± 0.04	0.033 ± 0.01	0.316 ± 0.05	0.731 ± 0.06	0.076 ± 0.02	0.053 ± 0.01
**30**	0.039 ± 0.01	0.025 ± 0.01	0.783 ± 0.49	0.083 ± 0.03	0.052 ± 0	0.017 ± 0
**Primary alcohols**						
**21**	0.002 ± 0	0	0.202 ± 0.06	0.069 ± 0.02	0	0
**22**	0.001 ± 0	0.004 ± 0	0.055 ± 0.01	0.698 ± 0.13	0.008 ± 0	0.007 ± 0
**23**	0.004 ± 0	0.001 ± 0	0.01 ± 0	0.788 ± 0.24	0.001 ± 0	0.001 ± 0
**24**	0.049 ± 0.01	0.002 ± 0	0.143 ± 0.04	0.941 ± 0.11	0.032 ± 0	0.11 ± 0.01
**25**	0.014 ± 0	0.003 ± 0	0.028 ± 0.01	0.1 ± 0.03	0.007 ± 0	0.017 ± 0
**26**	1.025 ± 0.07	0.025 ± 0	0.894 ± 0.18	2.734 ± 0.19	0.35 ± 0.02	0.786 ± 0.09

Compound	*S. canadensis*	*P. indica*	*F. chiloensis*	*R. nutkana*	*R. armeniacus*	*R. spectabilis*
**Primary alcohols**						
**27**	0.036 ± 0	0.002 ± 0	0.032 ± 0	0.108 ± 0.03	0.021 ± 0.01	0.013 ± 0
**Primary alcohols**						
**28**	0.392 ± 0.1	0.035 ± 0.01	0.561 ± 0.06	1.06 ± 0.12	1.142 ± 0.11	0.313 ± 0.02
**29**	0.011 ± 0	0.006 ± 0	0.006 ± 0	0.06 ± 0.01	0.019 ± 0	0.013 ± 0
**30**	0.107 ± 0	0.006 ± 0	0.11 ± 0.01	0.05 ± 0.01	0.605 ± 0.06	0.2 ± 0.01
**32**	0.293 ± 0.04	0.021 ± 0.01	0.19 ± 0.1	0.111 ± 0.02	0.17 ± 0.1	0.178 ± 0.02
**Esters**						
**38**	0.158 ± 0.01	0.027 ± 0.01	0.246 ± 0.07	0.185 ± 0.03	0.197 ± 0.1	0.004 ± 0
**40**	0.035 ± 0	0.006 ± 0	0.288 ± 0.09	0.839 ± 0.11	0.072 ± 0.01	0.011 ± 0
**42**	0.039 ± 0	0.011 ± 0	0.968 ± 0.16	0.82 ± 0.25	0.245 ± 0.06	0.039 ± 0.01
**44**	0.179 ± 0	0.008 ± 0	1.589 ± 0.15	1.13 ± 0.16	0.721 ± 0.18	0.17 ± 0.04
**46**	0.117 ± 0.01	0.017 ± 0	1.392 ± 0.08	0.44 ± 0.03	1.601 ± 0.44	0.118 ± 0.02
**48**	0.051 ± 0	0.026 ± 0	1.05 ± 0.13	0.207 ± 0.02	2.08 ± 0.56	0.17 ± 0.04
**50**	0.018 ± 0	0.034 ± 0.01	0.523 ± 0.05	0.069 ± 0.01	2.123 ± 0.52	0.098 ± 0.03
**52**	0.005 ± 0	0.02 ± 0	0.154 ± 0.01	0.016 ± 0	0.929 ± 0.23	0.027 ± 0.01
**Triterpenoids**						
**Alpha amyrin**	0	0	0	0	0.408 ± 0.16	0
**Beta amyrin**	0	0	0	0	0.155 ± 0.08	0.157 ± 0.01
**Erythrodiol**	0	0	0	0.142 ± 0.02	0.083 ± 0.01	0.016 ± 0
**Oleanoic acid**	1.766 ± 0.12	0.038 ± 0.01	0.205 ± 0.02	0	0.097 ± 0.04	0.011 ± 0
**Ursolic acid**	1.491 ± 0.19	0.058 ± 0.01	0.249 ± 0.05	0.225 ± 0.08	0.574 ± 0.13	0.055 ± 0.01
**Uvaol**	0	0	1.204 ± 0.14	0.263 ± 0.04	0	0
**Unknowns**	2.484 ± 0.12	0.484 ± 0.11	5.59 ± 0.3	5.611 ± 0.63	4.339 ± 1.03	0.856 ± 0.19

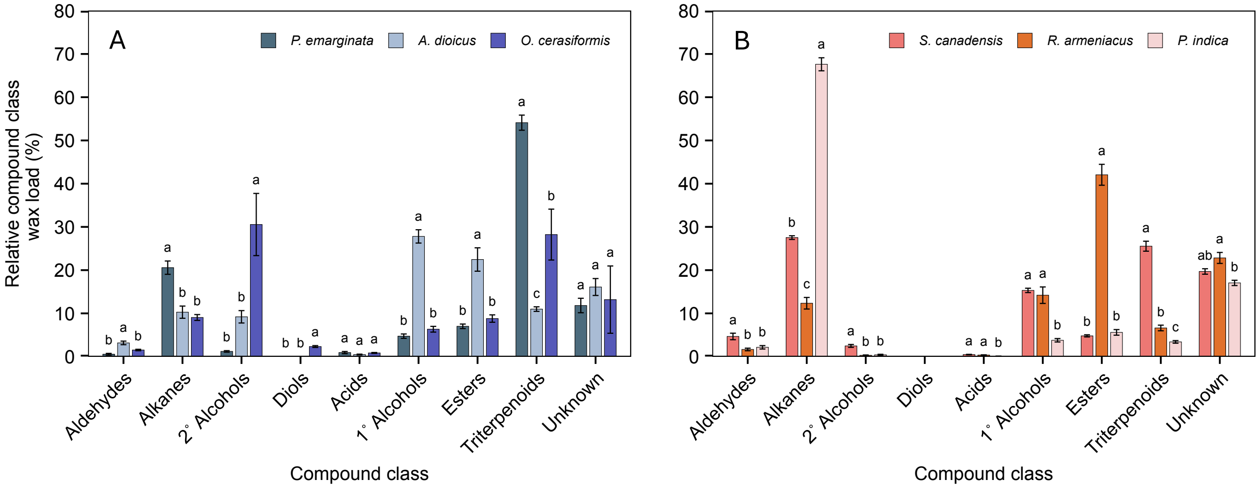

Fig. S2 Leaf cuticular wax composition of six Rosaceae species. Relative wax loads by compound class of Amygdaloideae (A) and Rosoideae (B) species. Bars represent the mean of biological replicates (n = 5), and error bars represent the standard error. Different letters represent significantly different relative compound class wax loads between species ($p < 0.05$, ANOVA).

Table S6 Relative loads of pooled chain lengths of wax compounds of six Amygdaloideae species. Relative chain length amounts summed across the aldehydes, alkanes, secondary alcohols, diols, fatty acids and primary alcohols. Values are the means of biological replicates (n = 5) with error represented as standard error.

Chain length (carbon number)	*M. fusca*	*A. alnifolia*	*O. cerasiformis*	*P. emarginata*	*S. lucida*	*A. dioicus*
**C20**	2.28 ± 0.36	0.98 ± 0.25	1.09 ± 0.24	1.63 ± 0.49	0.9 ± 0.1	16.2 ± 3
**C21**	0.56 ± 0.12	0	0	0.09 ± 0.09	0	0
**C22**	2.83 ± 0.74	0.57 ± 0.08	0.87 ± 0.16	1 ± 0.08	0.58 ± 0.06	5.04 ± 0.66
**C23**	0.29 ± 0.05	0	0.02 ± 0.01	0.67 ± 0.35	0.01 ± 0	0.05 ± 0.01
**C24**	7.91 ± 0.94	0.51 ± 0.05	2.66 ± 0.45	2.29 ± 0.25	1.47 ± 0.16	10.93 ± 1.55
**C25**	0.99 ± 0.12	0.16 ± 0.02	0.25 ± 0.05	1.44 ± 0.19	0.07 ± 0.01	4.03 ± 1.46
**C26**	11.75 ± 0.62	1.11 ± 0.08	2.17 ± 0.26	7.95 ± 0.55	0.72 ± 0.09	13.26 ± 1.71
**C27**	2.17 ± 0.25	2.34 ± 0.05	0.7 ± 0.11	4.41 ± 0.49	0.36 ± 0.07	5.79 ± 1.9
**C28**	21.25 ± 0.59	5.37 ± 0.39	5.18 ± 1.14	11.61 ± 3.44	3.49 ± 0.42	6.88 ± 0.48
**C29**	7.88 ± 0.72	79.65 ± 1.13	61.06 ± 7.79	26.54 ± 1.68	65.92 ± 1.48	7.12 ± 1.49
**C30**	9.71 ± 0.41	3.07 ± 0.33	10.07 ± 2.82	4.79 ± 0.63	13.95 ± 0.89	17.78 ± 2.53
**C31**	23.31 ± 1.94	5.09 ± 0.33	15.81 ± 2.94	32.05 ± 1.56	12.23 ± 0.77	9.09 ± 2.69
**C32**	8.56 ± 1.7	0.8 ± 0.11	0.12 ± 0.02	0.37 ± 0.16	0.22 ± 0.02	3.83 ± 0.54
**C33**	0.52 ± 0.07	0.36 ± 0.08	0	5.15 ± 1.31	0.1 ± 0.04	0

Table S7 Relative loads of pooled chain lengths of monomeric wax compounds of six Rosoideae species. Relative chain length amounts summed across the aldehydes, alkanes, secondary alcohols, diols, fatty acids and primary alcohols. Values are the means of biological replicates (n = 5) with error represented as standard error.

Chain length (carbon number)	*S. canadensis*	*P. indica*	*F. chiloensis*	*R. nutkana*	*R. armeniacus*	*R. spectabilis*
**C20**	0.06 ± 0.01	0	1.15 ± 0.3	0.53 ± 0.06	2.29 ± 0.23	2.53 ± 0.29
**C21**	0.03 ± 0	0	1.18 ± 0.4	0.27 ± 0.07	0	0
**C22**	0.11 ± 0.02	0.69 ± 0.17	1.13 ± 0.35	2.94 ± 0.38	0.75 ± 0.26	0.89 ± 0.15
**C23**	0.05 ± 0	0.05 ± 0.05	0.05 ± 0.02	3.03 ± 0.98	0.03 ± 0.01	0.05 ± 0.02
**C24**	2.39 ± 0.11	1.59 ± 0.4	2.69 ± 0.79	4.54 ± 0.5	1.64 ± 0.22	5.83 ± 0.37
**C25**	0.51 ± 0.03	0.4 ± 0.08	0.48 ± 0.08	1.21 ± 0.15	0.66 ± 0.16	1.24 ± 0.18
**C26**	21.33 ± 0.2	2.13 ± 0.36	7.55 ± 1.43	15.16 ± 0.59	8.53 ± 0.6	37.87 ± 2.7
**C27**	1.47 ± 0.1	2.68 ± 0.95	1.86 ± 0.19	2.99 ± 0.17	1.45 ± 0.11	3.3 ± 0.49
**C28**	12.49 ± 1.66	3.72 ± 0.38	6.86 ± 0.6	12.02 ± 0.49	27.08 ± 1.61	18.44 ± 0.61
**C29**	0.66 ± 0.06	4.35 ± 0.51	1.38 ± 0.16	4.43 ± 0.2	3.95 ± 0.78	6.48 ± 1.31
**C30**	3.07 ± 0.2	3.01 ± 0.12	7.37 ± 2.39	1.33 ± 0.1	20.2 ± 0.65	11.43 ± 0.46
**C31**	14.12 ± 0.81	36.11 ± 0.8	30.68 ± 1.98	25.02 ± 0.85	4.87 ± 1.07	3.34 ± 1.27
**C32**	4.57 ± 0.52	3.06 ± 0.21	2.12 ± 0.66	0.87 ± 0.1	28.57 ± 1.96	8.05 ± 0.92
**C33**	39.14 ± 0.84	42.21 ± 2.22	35.5 ± 1.57	25.68 ± 1.17	0	0.56 ± 0.16

Table S8 Average chain length of wax compound classes. Values are the means of biological replicates (n = 5) with error represented as standard error.

Species	Aldehydes	Alkanes	Secondary alcohols	Diols	Acids	Primary alcohols	Esters
M. fusca	28.6 ± 0.09	30.1 ± 0.07	29.53 ± 0.21		25.11 ± 0.32	27.71 ± 0.2	45.76 ± 0.25
A. alnifolia	29.59 ± 0.02	29.15 ± 0.04	29.05 ± 0.01	29	25.67 ± 0.22	28.16 ± 0.05	41.88 ± 0.27
O. cerasiformis	29.1 ± 0.08	29.51 ± 0.07	29.58 ± 0.32	29	26.45 ± 0.28	27.42 ± 0.22	42.96 ± 0.13
P. emarginata	28.81 ± 0.38	29.96 ± 0.05	30.89 ± 0.03		25.65 ± 0.54	26.61 ± 0.16	44.76 ± 0.2
S. lucida	29.84 ± 0.03	30.11 ± 0.04	29.15 ± 0.01	29	25.71 ± 0.28	29.02 ± 0.09	41.99 ± 0.11
A. dioicus	28.58 ± 0.12	28.28 ± 0.41	29.48 ± 0.37		21.45 ± 0.29	26.81 ± 0.3	46.01 ± 0.13
S. canadensis	27.28 ± 0.04	32.36 ± 0.02	31 ± 0.08		26.49 ± 0.16	27.48 ± 0.09	43.03 ± 0.09
P. indica	26.79 ± 0.42	31.74 ± 0.1	29.72 ± 0.13		27.37 ± 0.1	27.99 ± 0.25	45.86 ± 0.47
F. chiloensis	27.67 ± 0.08	31.82 ± 0.05	31.26 ± 0.13		26.04 ± 0.83	26.68 ± 0.45	45.09 ± 0.16
R. nutkana	27.4 ± 0.03	31.4 ± 0.05	31.81 ± 0.06		26.14 ± 0.11	25.38 ± 0.1	42.98 ± 0.09
R. armeniacus	28.64 ± 0.11	31.11 ± 0.1	27.98 ± 0.04		24.55 ± 0.18	28.39 ± 0.17	47.73 ± 0.22
R. spectabilis	27.03 ± 0.17	29.13 ± 0.21	29.16 ± 0.26	29	24.62 ± 0.14	27.41 ± 0.11	46.39 ± 0.15

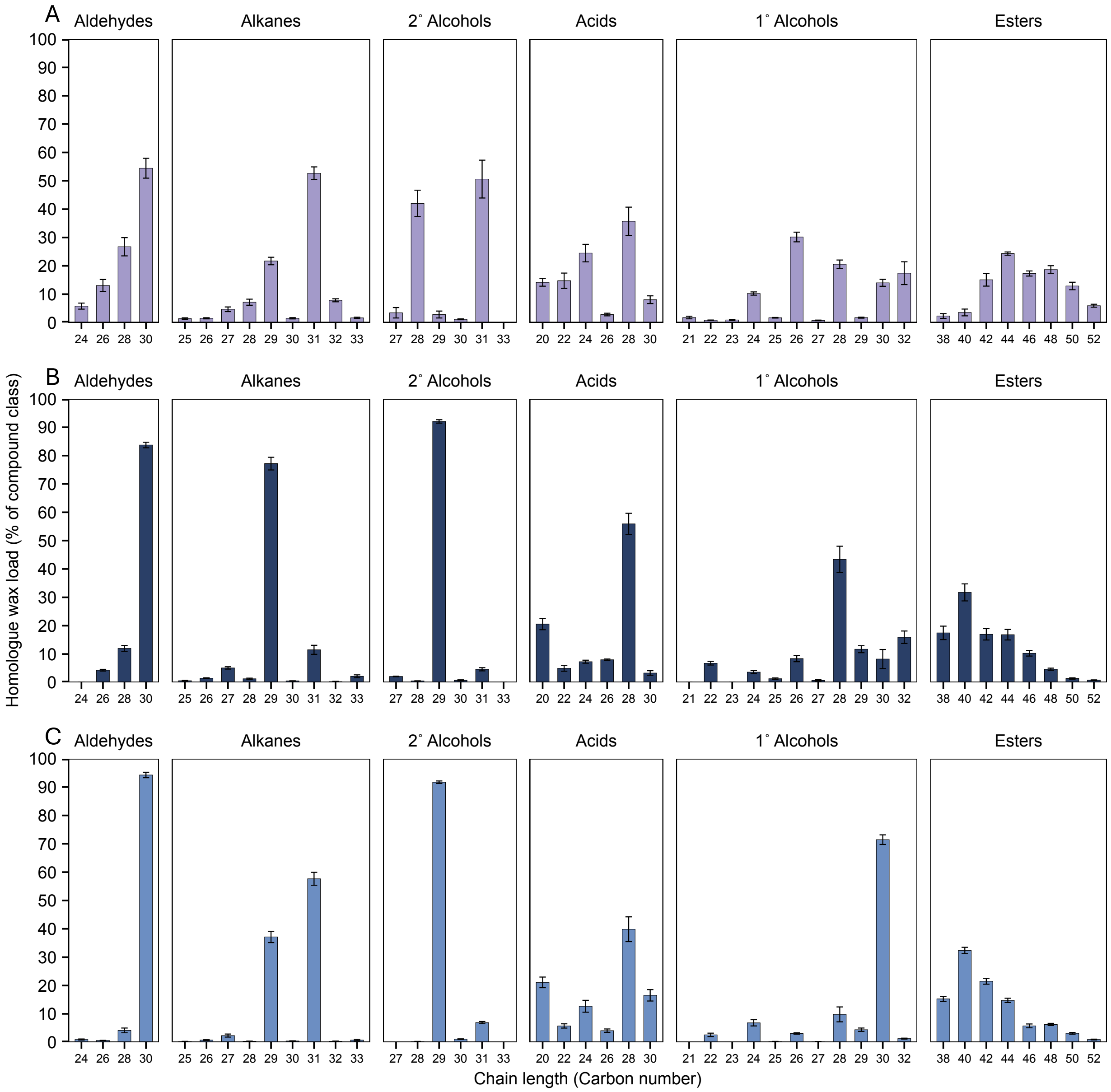

Fig. S3 Homologue distribution of wax compound classes in three main Amygdaloideae species. Relative contribution of each homologue detected in compound classes in (A) *Malus fusca*, (B) *Amelanchier alnifolia*, and (C) *Spiraea lucida*. Bars represent the mean of biological replicates (n = 5), and error bars represent the standard error.

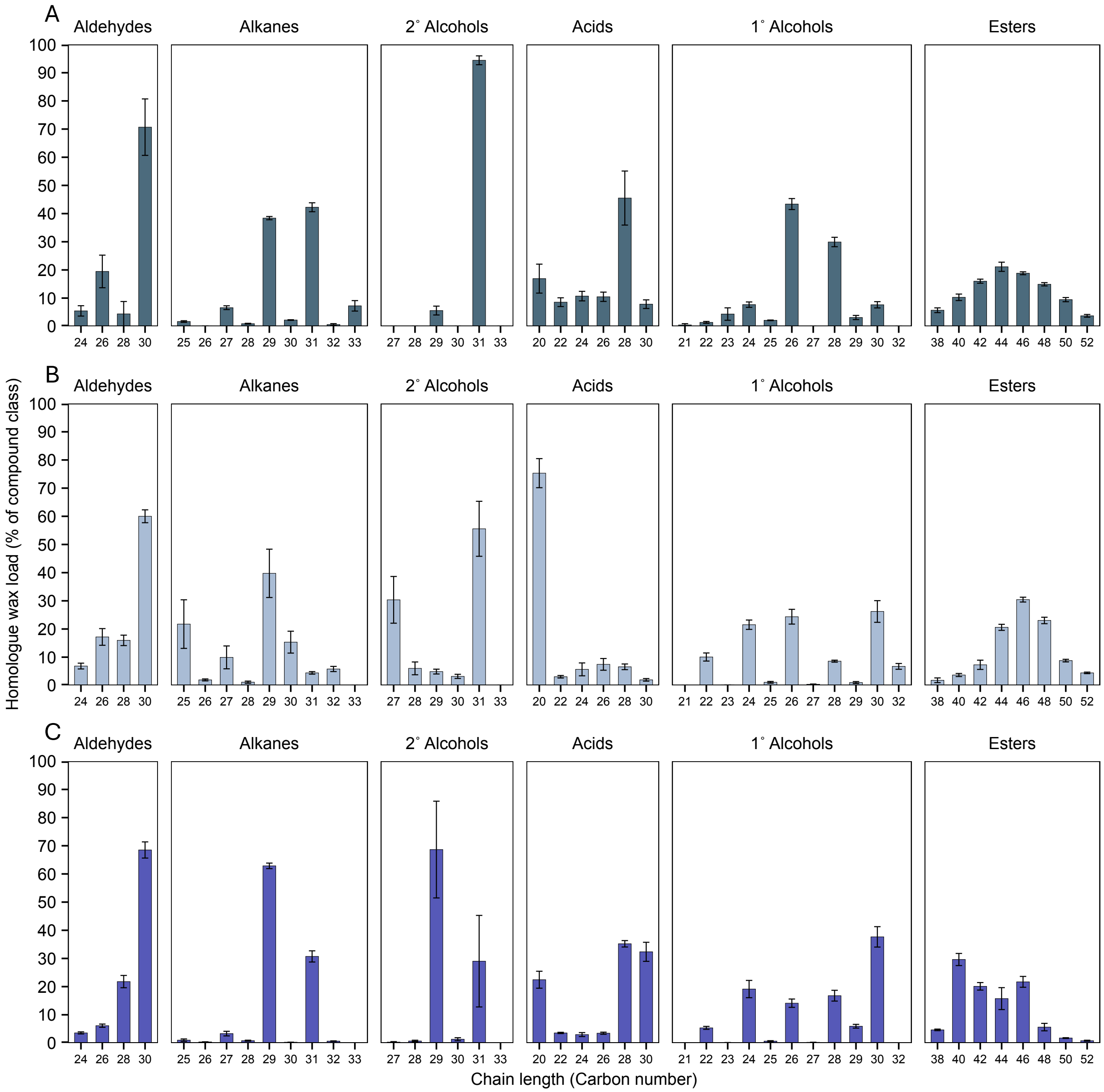

Fig. S4 Homologue distribution of wax compound classes in three supplemental Amygdaloideae species. Relative contribution of each homologue detected in compound classes in (A) *Prunus emarginata*, (B) *Aruncus dioicus*, and (C) *Oemleria cerasiformis*. Bars represent the mean of biological replicates (n = 5), and error bars represent the standard error.

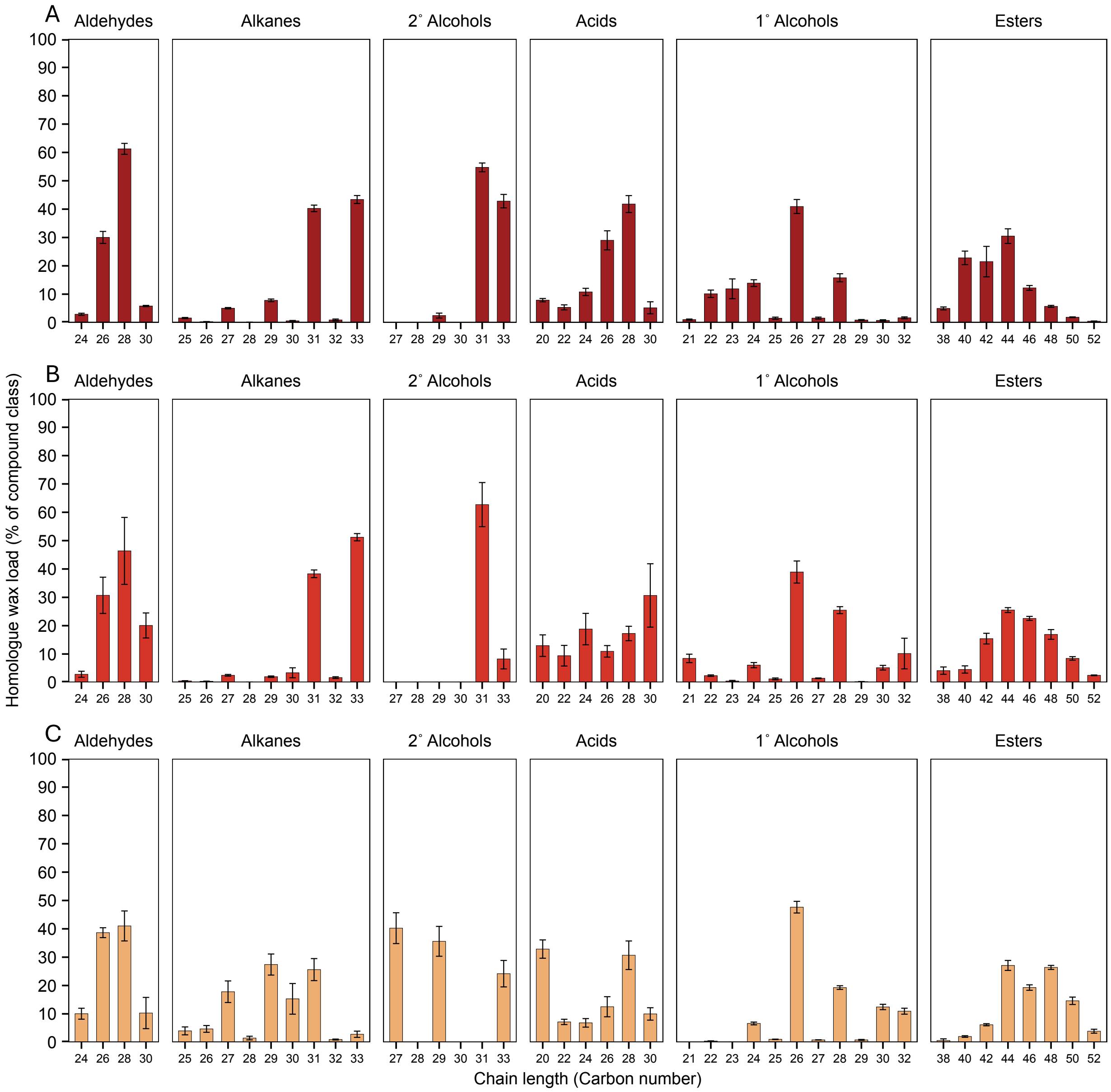

Fig. S5 Homologue distribution of wax compound classes in three main Rosoideae species. Relative contribution of each homologue detected in compound classes in (A) *Rosa nutkana*, (B) *Fragaria chiloensis*, and (C) *Rubus spectabilis*. Bars represent the mean of biological replicates (n = 5), and error bars represent the standard error.

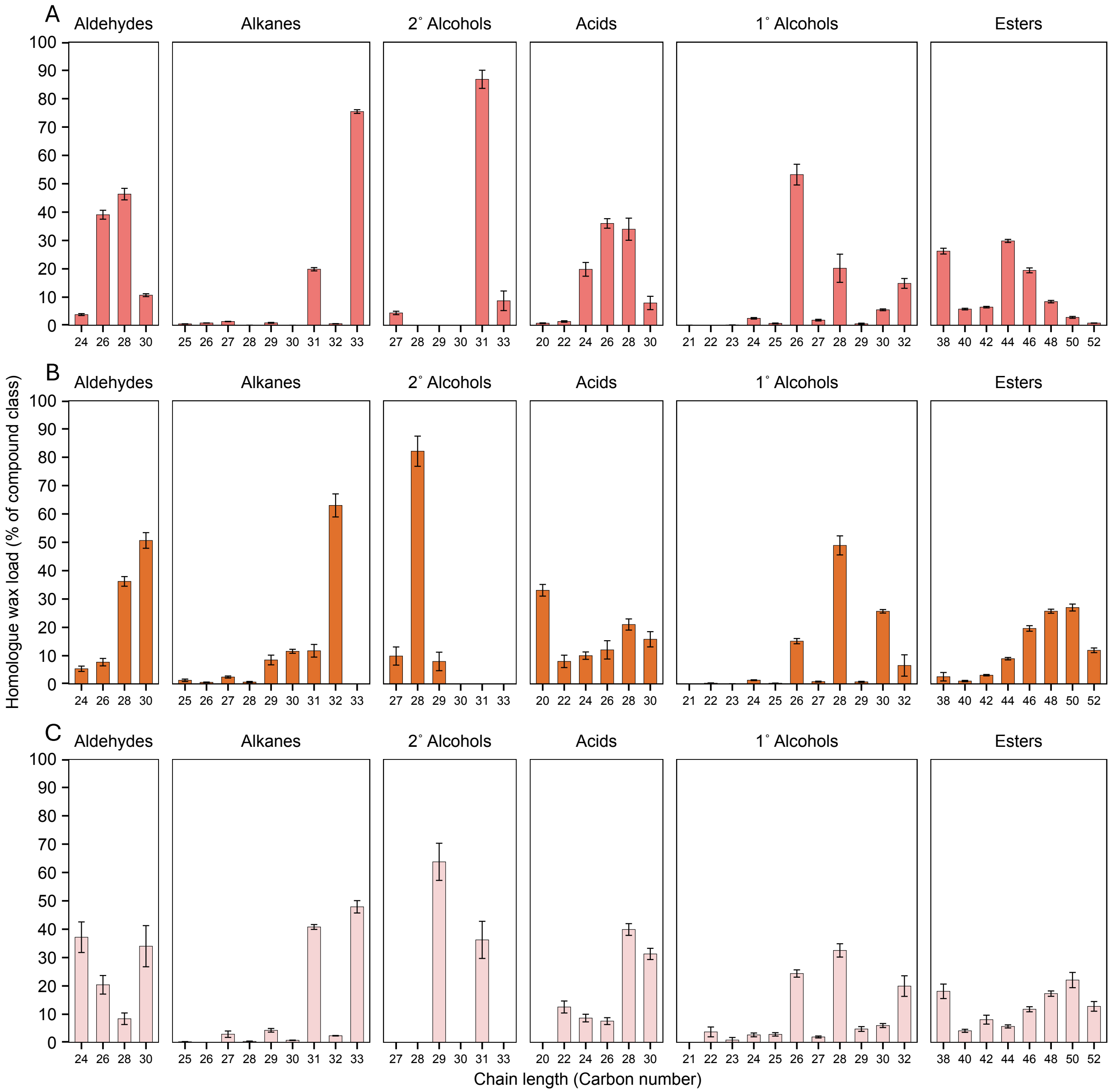

Fig. S6 Homologue distribution of wax compound classes in three supplemental Rosoideae species. Relative contribution of each homologue detected in compound classes in (A) *Sanguisorba canadensis*, (B) *Rubus armeniacus*, and (C) *Potentilla indica*. Bars represent the mean of biological replicates (n = 5), and error bars represent the standard error.

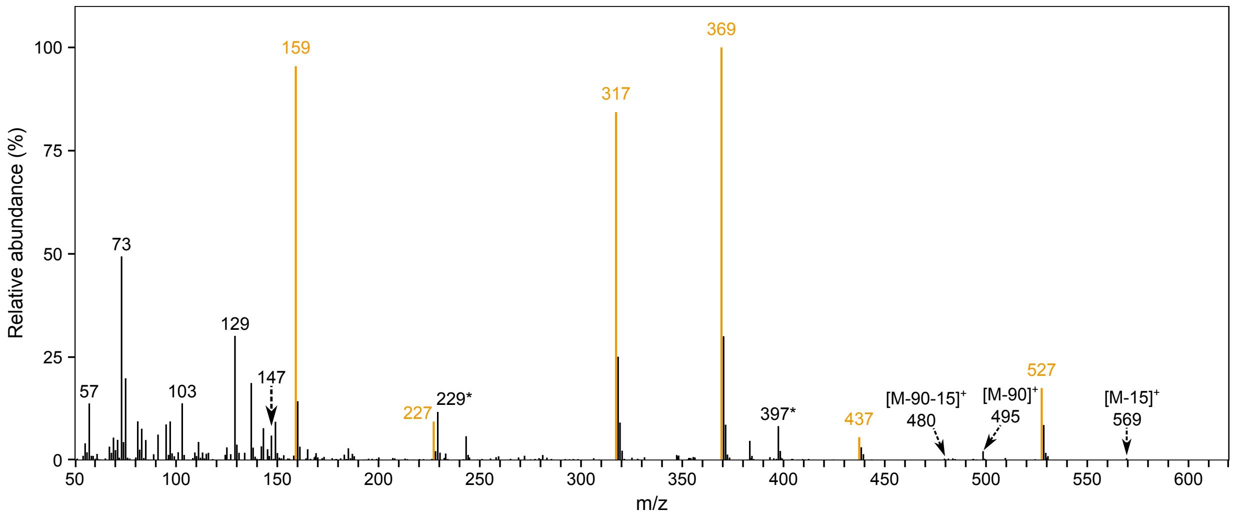

Fig. S7 GC-MS identification of nonacosane-5,10-diol. Mass spectrum and fragmentation diagram (top right) of *Amelanchier alnifolia* nonacosane-5,10-diol (TMS-ether); characteristic fragment ions are highlighted. * Indicates characteristic α-fragments of co-eluting 10-hentriacontanol that could not be resolved from spectrum.

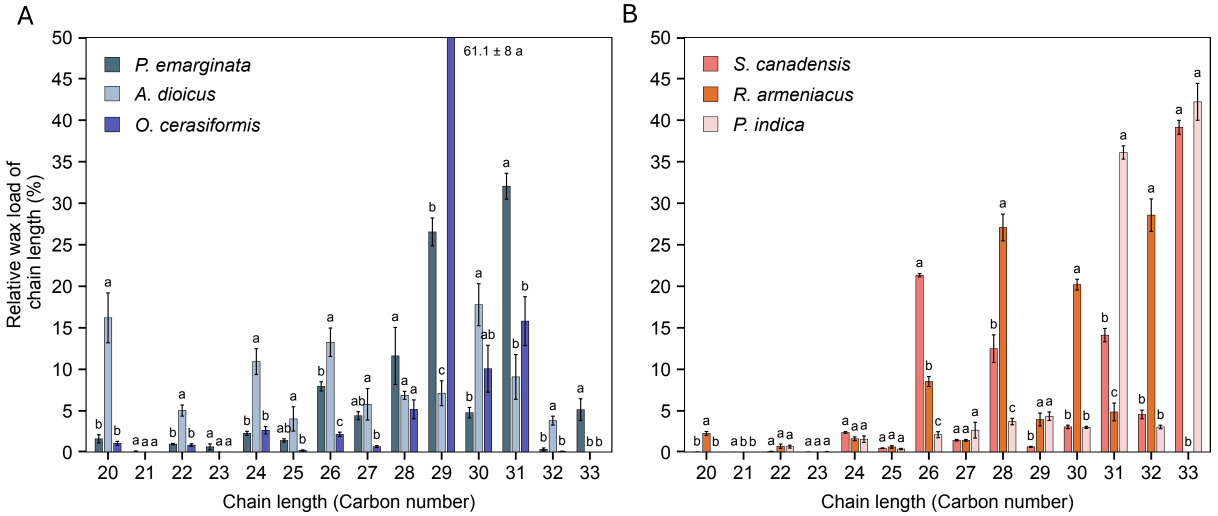

Fig. S8 Pooled chain length distribution of cuticular wax compounds for six Rosaceae species. Relative chain length amounts summed across the aldehydes, alkanes, secondary alcohols, diols, fatty acids and primary alcohols in the leaf wax mixtures of Amygdaloideae (A) and Rosoideae (B) species. Bars represent the mean of biological replicates (n = 5), and error bars represent the standard error. Different letters represent significantly different relative chain length wax loads between species ($p < 0.05$, ANOVA).

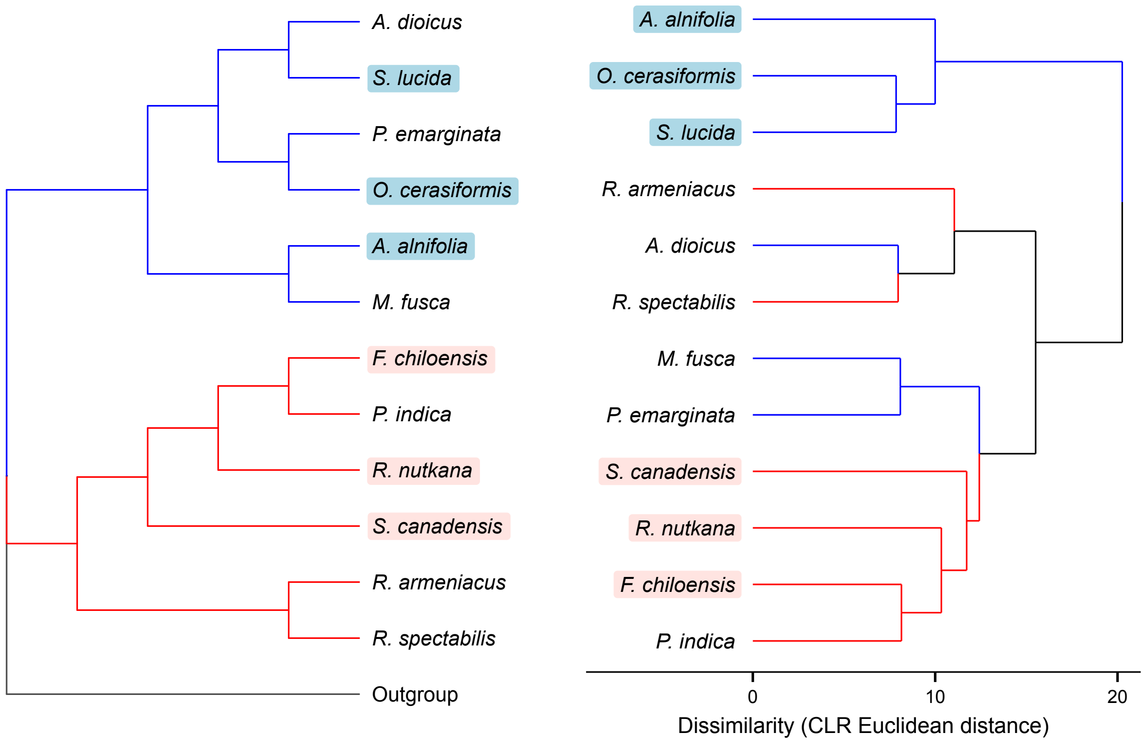

Fig. S9 Comparison of Rosaceae phylogeny to hierarchical clustering based on Rosaceae wax composition. Cladogram of studied Rosaceae generated from rbcL sequences (left) aligned with hierarchical clustering dendrogram generated from centred log-ratio (CLR) transformed relative individual wax compound amounts (right). Blue and red lines denote members of the Amygdaloideae and Rosoideae subfamilies, respectively. Blue and pink highlighting indicate species with epicuticular wax tubules and irregular platelets, respectively.
